# Multidimensional kinetic study on the organocatalyzed ring-opening polymerization (ROP) of l-lactide *via* a robotic high-throughput flow platform

**DOI:** 10.1039/d5sc03998c

**Published:** 2025-12-26

**Authors:** Bo Zhang, Tanja Junkers

**Affiliations:** a Polymer Reaction Design Group, School of Chemistry, Monash University 17 Rainforest Walk Clayton VIC 3800 Australia tanja.junkers@monash.edu

## Abstract

Ring-opening polymerization (ROP) of l-lactide catalyzed by 1,5,7-triazabicyclo[4.4.0]dec-5-ene (TBD) was efficiently screened *via* a programmable high-throughput robotic flow platform with very high accuracy (absolute error <5%). With a very significant amount of data being generated, a rate law describing living ROP initiated by TBD that includes a first-order dependency in l-lactide, a first-order dependency in TBD and a half-order dependency in the initiator 4-methylbenzyl alcohol is developed. Additionally, a negative observed reaction rate (*k*_obs_) was determined with increasing the initial monomer concentration, initiator or catalyst, or, surprisingly, decreasing the reaction temperature, giving rise to negative activation energies within the chemical space studied.

## Abstract 2

The ring-opening polymerization (ROP) of l-lactide catalyzed by TBD was efficiently studied using a high-throughput robotic flow platform with very high accuracy, and a rate law of the reaction was revealed based on a comprehensive kinetic study.

## Graphical abstract

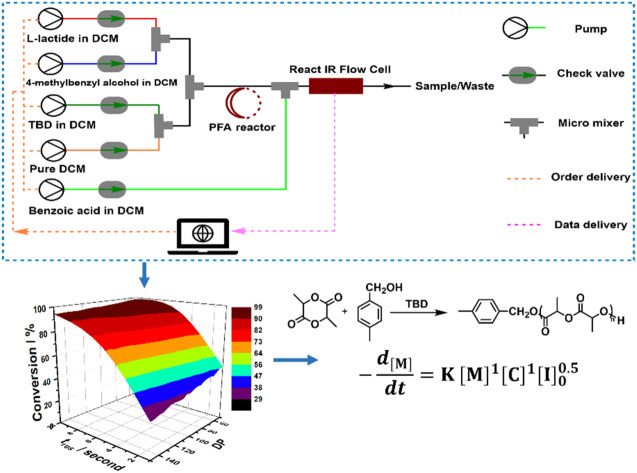

## Introduction

Ring-opening polymerization (ROP) is one of the most powerful techniques in the realm of controlled polymerization, enabling the synthesis of well-defined and often biodegradable polymers with a wide range of lengths, compositions, and architectures. Since the first use of 4-dimethylaminopyridine in ROP, organic catalysts have developed to a high sophistication, offering simpler and more durable alternatives to their metal–organic counterparts.^1^ Their ease of purification, insensitivity to oxygen, adaptability to various conditions and straightforward removal from polymers provide significant advantages in polymer synthesis.^2–4^ Specifically, ROP catalyzed by 1,5,7-triazabicyclo[4.4.0]dec-5-ene (TBD) offers a rare combination of narrow molecular weight distributions at very high monomer conversions and fast polymerization rates.^5,6^ This is the result of a rapid initiation step, facilitated by the formation of a monomer-catalyst-initiator intermediate through hydrogen bonding, and the absence of undesired termination reactions.^7–9^ ROP catalyzed by TBD has been employed to synthesize various polyesters such as polylactide and polycaprolactone that have given rise to promising applications in the medical field,^10–12^ or for example to sequence-controlled polymers.^13,14^ Despite those synthetic advancements, the kinetics of organocatalyzed ROP remains, however, relatively unexplored because the reaction proceeds so rapidly that obtaining sufficient usable data for kinetic studies is extremely challenging, leading to reports of only sparse and poorly reproducible data.^5,15–18^

Flow chemistry has drawn significant attention in the field of polymer synthesis in recent years due to the excellent heat and mass transfer of the flow reactors, which allows them to conduct reactions under nearly ideal thermodynamic conditions. This significantly reduces batch-to-batch variations and enhances the reproducibility and predictability of the reactions, making them ideal for kinetic studies.^19,20^ Flow reactions are also highly automatable. With the integration of machine automation and online/inline analysis techniques, human bias associated with altering experimental parameters, quenching reactions after a set residence time, and preparing samples for analysis is eliminated, thus further expanding the operation window and increasing the reliability of generated data.^21,22^ With the advantages mentioned above, flow chemistry has achieved great success in reaction screening and precise polymer synthesis.^13,22–26^ Our group recently achieved the screening of multidimensional kinetic profiles of radical polymerization reactions with full conversion on a minute scale *via* an automated high-throughput robotic flow platform with excellent accuracy.^27^

**Scheme 1:**
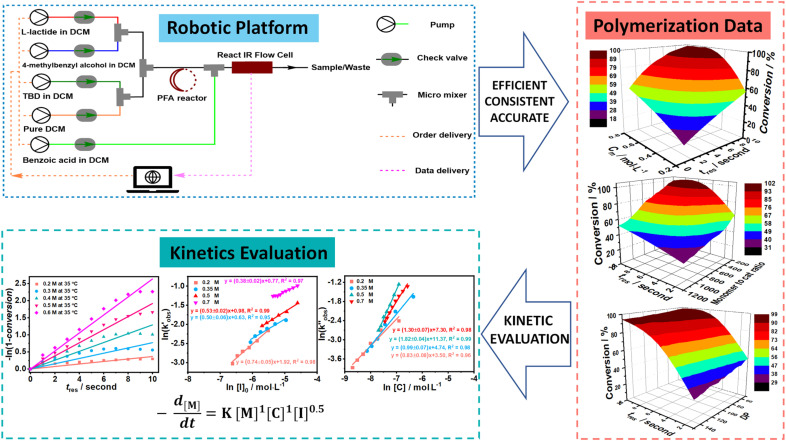
Overview of multidimensional kinetic profile screening and kinetics studying *via* the programmable high-throughput robotic flow platform.

Yet, even with the above-mentioned capability, the ultrafast ROP that yields full conversions on a lower second timescale remains a formidable challenge with respect to time-resolved kinetic studies and analysis. Waymouth and colleagues reported a class of urea anion catalysts that are highly active and selective in the ring-opening polymerization (ROP) of cycloesters, achieving 100% conversion in just seconds. However, due to limitations in monitoring techniques and fast polymerization of lactide, only the kinetics of ε-caprolactone, catalyzed by a relatively slower catalyst, were studied, with full conversion reached in approximately 13 minutes.^28^ Two years later, his team developed a sophisticated automated flow synthesizer towards ROP, yet always pushing the conversion of cyclic esters to 100% to rapidly generate block copolymer libraries. Thus, their focus was primarily on materials library construction, leaving the kinetic aspects unexplored.^3^ Although several studies have explored the rate laws of the ROP of l-lactide, they have primarily focused on relatively slow polymerization systems, such as those catalyzed by zinc complex,^29,30^ tin(ii) octoate^31–36^ or N-heterocyclic molecules^37^ at high temperature (higher than 100 °C), achieving full conversion in hours. Furthermore, most of these studies only address the influence of monomer and initiator on the rate law, neglecting the catalyst's effect. l-lactide polymerization kinetics involving fast organocatalysts is thus rarely touched. Herein, we report an in-depth kinetic study of the ROP catalyzed by TBD based on the multidimensional kinetic profiles obtained from a further optimized programmable robotic flow platform (as presented in Scheme 1). With this platform, the monomer, initiator and catalyst concentration can be automatically changed independently from each other, and also the overall dilution level of the mixture can be adjusted dynamically. A step-change reaction solution was generated using four streams of stock solutions (monomer, catalyst, initiator, and pure solvent), all controlled by a pre-defined Python script. To adapt the platform for screening reactions at low initial monomer concentrations (less than 1 M in the ROP of l-lactide system), where significant systematic errors (around 30%) were observed in our previous study,^27^ parallel experiments were systematically conducted to reduce statistical error, accompanied by an increase in the total flow rate to 2 mL min^−1^. The solution was then delivered to the reactor, where polymerization took place. To minimize the impact of residence time fluctuations on the ROP conversion, inline quenching was introduced to terminate the reaction as soon as the product eluted from the reactor. Additionally, an inline FT-IR was installed right after the quench point to analyze the products efficiently and accurately. With this, we were able to collect a very substantial amount of data with excellent accuracy (absolute error <5%) and high time efficiency. This ‘big data’ collection enabled us to derive rate laws for the living ROP of l-lactide catalyzed by TBD, revealing a negative observable activation energy ((*E*_a_)_obs_).

## Results and discussion

Our kinetic study of TBD catalyzed ROP started by screening its kinetic profiles with the optimized automated robotic platform. The l-lactide ring breathing mode peak, ranging from 940 to 928 cm^−1^, was followed on the IR spectra,^38^ and based on the pre-built calibration curve, as shown in Fig. S4(B), the residue monomer concentration was automatically calculated from a Python script.

**Fig. 1:**
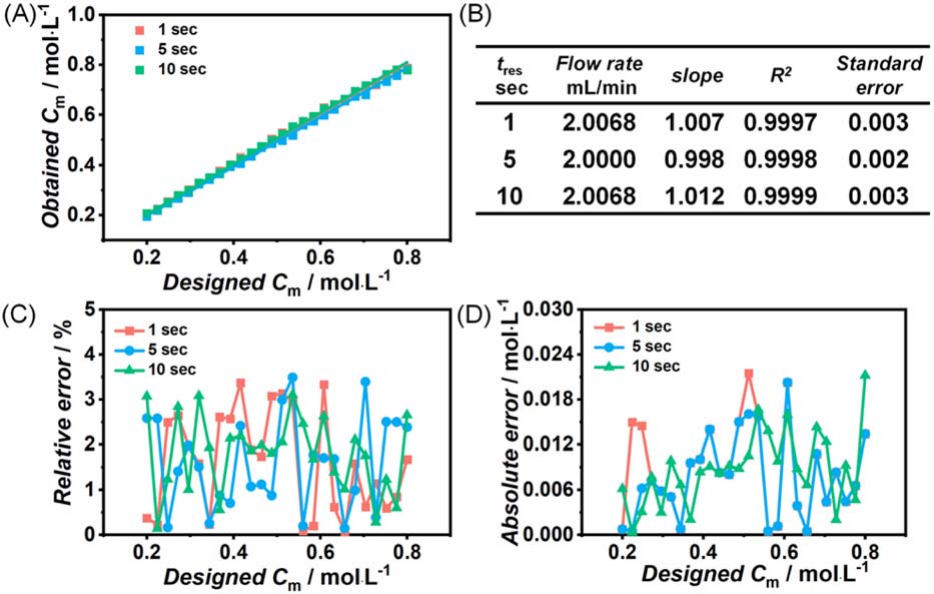
(A) Experimentally obtained monomer concentration from inline FT-IR *vs.* the ideal input concentration gradient; (B) fit statistics for (A); (C) relative error, and (D) absolute error between the designed and experimental monomer concentration.

Before starting screening, the accuracy of the platform was tested by only programming concentration step traces (monomer concentration ramping from 0.2 M to 0.8 M). To simplify, stock solutions of raw materials were prepared, and the streams for TBD and benzoic acid were swapped to avoid polymerizations. 3 syringe pumps were used to deliver the stock solution of monomer (with 100 to 1 molar equivalent of initiator), TBD and pure DCM and a peristaltic pump was applied to deliver the stock solution of benzoic acid to generate a monomer concentration of gradient ranging from 0.2 to 0.8 M while keeping the monomer-to-catalyst ratio fixed at 200 (see Scheme S1(B)). The outcome of these test flow conditions is given in Fig. 1. Fig. 1(A) depicts programmed concentration against the measured concentration, and the linearity nicely confirms the validity of the approach and accuracy of the concentration sweeping, even at concentrations lower than 1 M. Fitting results are given in Fig. 1(B). Both the fitting slope and the *r*^2^ value of the fit are very close to one, with the standard error being smaller than 0.003, indicating the reaction solution generated from the optimized platform closely matches our design specifications. Given the high speed of the screening and the short overall residence times, this is a very satisfying result. Closer inspection of the error is given in Fig. 1(C) and (D), for the relative and absolute error between the designed monomer concentration and the determined concentration during the sweep. The relative error remains relatively constant, below 4%, while the absolute error between the experimental and intended monomer concentrations remains below 0.02 M on a 1 to 10 seconds time scale. These improvements are significant compared to the previous values of 30% and 0.1 M in our previous iteration of the platform.^27^ Thus, the optimized robotic platform exhibits excellent accuracy in screening second-scale polymerizations and for comparatively lower monomer concentrations, marking a distinct advancement that can now be exploited for the study of very fast reactions. With the platform being validated, comprehensive screening experiments were conducted, including monomer concentration sweeping experiments, monomer-to-catalyst ratio sweeping experiments and degree of polymerization sweeping experiments (by changing the monomer to initiator concentration ratio). These experiments covered almost all dimensions that can influence the ROP, including monomer concentration, residence time, temperature, initiator concentration and catalyst concentration. The overview of the sweeping platform and flow rate of different streams for different experiments is given in Scheme S1 and Fig. S1–S3, respectively. Duplicate experiments were conducted to validate the reproducibility of the robotic platform. As can be seen from the conversions of repeat experiments shown in Fig. S6–S14, the ROP of l-lactide catalyzed by TBD almost 100% monomer conversion is consistently achieved within 10 seconds or faster. Data from duplicate experiments align closely, further demonstrating the excellent reproducibility of the robotic platform for these ultrafast polymerizations. As a last confirmation of the high accuracy, we also benchmarked the IR data against high-field NMR conversion determinations, which also demonstrated excellent accuracy with only a 5% deviation (Tables S2–S4).

**Fig. 2:**
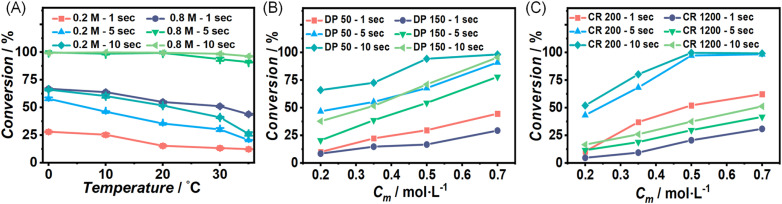
Summary of conversion data from (A) concentration-sweep experiments carried out at different temperatures with error bars indicating the difference from duplicate experiments, (B) degree of polymerization sweeping experiments conducted at different initial monomer concentrations and (C) monomer to catalyst (catalyst ratio CR) sweeping experiments performed at different initial monomer concentrations. All conversion data were calculated from inline IR.

Furthermore, consistent conversion trends were observed as summarized in the sweeping experiments shown in Fig. 2. Fig. 2(A) presents the results generated from concentration-sweep experiments and shows the counterintuitive trend of monomer conversion decreasing with increasing temperature. This observation is no artifact, and as noted above well reproducible. And indeed, when increasing the residence time or the initial monomer concentration, increases in monomer conversion are observed with errors between duplicates of less than 5%. Data generated from degree of polymerization (DP) sweep experiments is demonstrated in Fig. 2(B), and a positive correlation between monomer conversion and initiator concentration is highlighted. Fig. 2(C) illustrates the conversions from monomer-to-catalyst ratio (CR) sweep experiments and reveals an increase in monomer conversion with increasing catalyst concentration, as one would expect.

The ROP of l-lactide catalyzed by TBD is generally believed to proceed *via* a hydrogen bonding mechanism according to theoretical studies, where an ‘intermediate’ of monomer, catalyst and initiator is first formed *via* hydrogen bonds, followed by the ring opening of monomer and the release of catalyst.^6,7,9,39^ The formation of the intermediate is identified as an exothermic reaction in rice's computational simulations study, exhibiting an energy barrier of 13.3 kcal mol^−1^.^9^ The data in Fig. 2 support this mechanism, as increased concentrations of monomer, initiator, and catalyst appear to accelerate intermediate formation, ultimately increasing monomer conversion. The peculiar apparent negative activation energy of the reaction is, however, not intuitive. It may, however, be understood by increasing the temperature, weakening the hydrogen bonds, thus disfavouring the formation of the intermediate.^40–42^ While data on the temperature dependence in ROP is somewhat scarce, similar reports have been made before, some indication for this behaviour has been found before in TBD-catalyzed ROP of lactide by Wennekes (even though some other data in this work pointed to other directions)^15^ or in a milder system, the bulk ROP polymerization of δ-decalactone, catalyzed by TBD by Hillmyer and coworkers.^43^

Next to these first kinetic insights, it is also important to check on the obtained molecular weights and dispersities, see Table S2 and Fig. S15 for information on samples collected at the end of concentration sweep experiments. The molecular weight of the polymer increases with both initial monomer concentration and residence time. The dispersity of the obtained polymer is relatively high due to transesterification side reaction at high conversions and the residence time distribution inside the non-ideal plug flow system,^8,44–46^ which, however, doesn't influence the propagation of the monomers and hence the rate of polymerization. Since this is a kinetic study, polymer dispersity is of lesser focus, thus, we did not concern ourselves with dispersity optimizaton.

The screening plots from sweeping experiments, as outlined in Fig. 2, however, give only small snapshots of the obtained data. After combining all data, full 3-dimensional representations can be built, see Fig. S16–S18 for kinetic screening for different sweeping experiments. Examples of these 3D plots for each system are also provided in the ‘Polymerization Data’ section in Scheme 1. With the reaction automation, it only takes around 0.5 h to collect all data for one of the shown 3D surface plots, which is, to the best of our knowledge, the fastest and most efficient method reported to date for such detailed screening of such fast polymerizations. Apart from the time advantage, the obtained data density is also very high, increasing the number of available data points for later kinetic analysis. For each of the 3D plots, a clear view is demonstrated of how monomer conversion is influenced by the parameters in the *X* (initial monomer concentration for concentration sweep experiments, residence time for degree of polymerization and monomer-to-catalyst ratio sweep experiments) and *Y* (residence time for concentration sweep experiments, monomer-to-catalyst ratio for monomer-to-catalyst ratio sweep experiments, and degree of polymerization for DP sweep experiments) axes, allowing to pick optimal reaction conditions for future experiments directly. To reach a functional description of the kinetic data, polynomial fitting was applied to fit the 3-dimensional surface plots originated from the experimental data (Fig. S16–S18). 3rd-degree polynomial fitting (eqn (S10)) was carried out *via* a Python script, and the fitting results are presented in Tables S5, S11 and S16 for concentration-sweeping experiments, monomer-to-catalyst ratio sweeping experiments and degree of polymerization sweeping experiments, respectively. *R*^2^ values of all fittings are very close to unity, underpinning the low scatter in the data. With the multi-dimensional profiles being built, the 3D data can now be sliced as needed, and kinetic analysis can be conducted with the interpolated data in any dimension on demand.

**Fig. 3:**
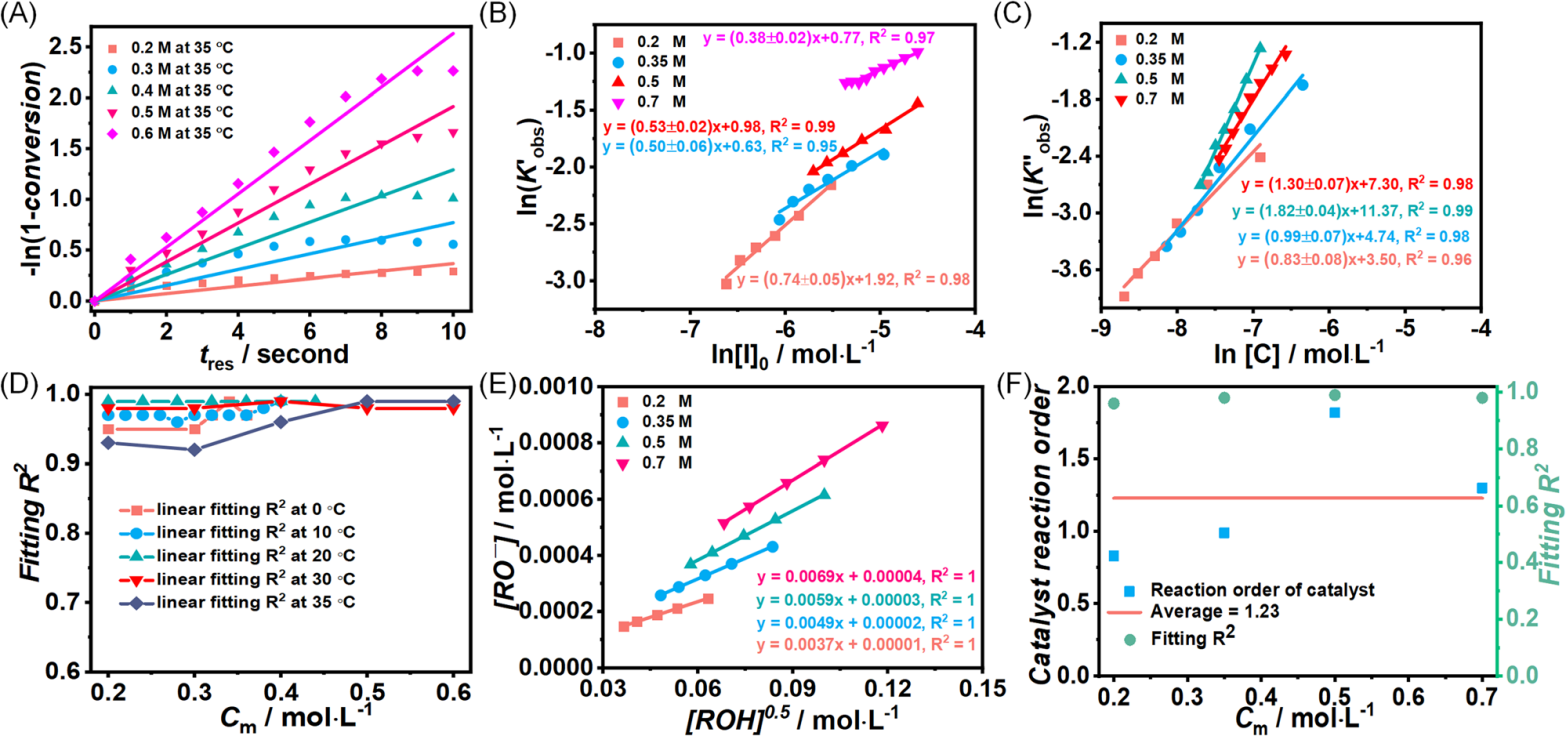
(A) Example plots of ln(*M*_0_*/M*_*t*_) *versus* residence time (*t*_res_)for the ROP of l-lactide and its summary (D). (B) Plots of 
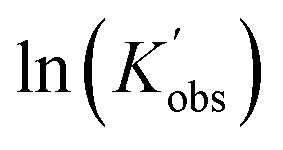
*versus* ln([*I*]_0_) for the ROP of l-lactide with different initial monomer concentrations, and Plots of the concentration of deprotonated alcohol ([RO^−^]) *versus* the square root of initial initiator concentrations ([ROH]^0.5^) at different initial monomer concentrations in acetonitrile (E). (C) Plots of 
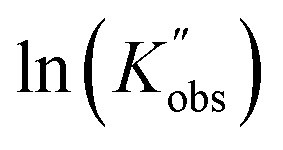
*versus* ln([*C*]) for the ROP of l-lactide with different initial monomer concentrations and the fitting summary (F). All the points presented here are derived from the data extracted from the original fitting. 
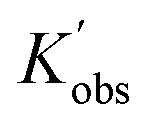
 is the observed rate constant generated from degree of polymerization sweeping experiments; 
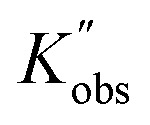
 is the observed rate constant generated from monomer-to-catalyst ratio sweeping experiments.

To better reveal the kinetics of ROP of l-lactide from our experimental data, the kinetic profiles (original 3-dimensional surface plots) built from the experimental data were sliced and studied (sliced data from the original 3-dimensional surface plots are presented in Tables S6–S10, S12–S15 and S17–S20). To reduce the dimensionality of the data and make it more processable, we continued our kinetic study by determining observable rate coefficients from all sweeping experiments. Observed rate coefficients from concentration sweeping experiments at different initial monomer concentrations were generated *via* fitting ln(*M*_0_*/M*_*t*_) against residence time. As an example of the generally high quality of fits, an example is given for 35 °C data in Fig. 3(A) (linear plots for other temperatures are provided in Fig. S19) with very good overall *R*^2^ values (as shown in Fig. 3(D) and summarized in Table S21). This underpins the well-controlled nature of the reactions. The monomer consumption remained first-order at all temperatures and initial monomer concentrations, and the rates of polymerization decreased with the lower monomer loading. The observed rate coefficients from DP sweeping were derived by fitting the ln(*M*_0_*/M*_*t*_) *versus* residence time at different combinations of initial monomer concentrations (0.2 to 0.7 mol L^−1^) and DPs (50 to 150), as presented in Fig. S20, and the fitting results were listed in Table S22. Fig. 3(B) summarizes the ln value of the observed rate coefficients 
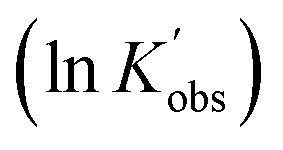
 against the ln values of initial initiator concentrations (ln[*I*]_0_) at different initial monomer concentrations (0.2, 0.35, 0.5 and 0.7 mol L^−1^). Similarly, the observed rate coefficients from monomer-to-catalyst ratio sweeping experiments were obtained *via* fitting the ln(*M*_0_*/M*_*t*_) *versus*. residence time at different combinations of initial monomer concentrations (0.2 to 0.7 mol L^−1^) and monomer-to-catalyst ratios (50 to 150), as shown in Fig. S21, and the fitting results were summarized in Table S23. The ln value of the observed rate coefficients 
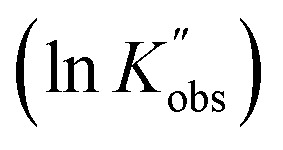
 against the ln values of catalyst concentrations (ln[*C*]) at different initial monomer concentrations (0.2, 0.35, 0.5 and 0.7 mol L^−1^) are given in Fig. 3(C).

Analysis of the ln(*M*_0_*/M*_*t*_) *versus* residence time revealed that TBD catalyzed ROP of l-lactide is first-order in monomer concentration. Having decided the reaction order of monomer, we proposed the overall rate law for the TBD catalyzed ROP polymerization of l-lactide initiated by 4-methylbenzyl alcohol, as shown below.1
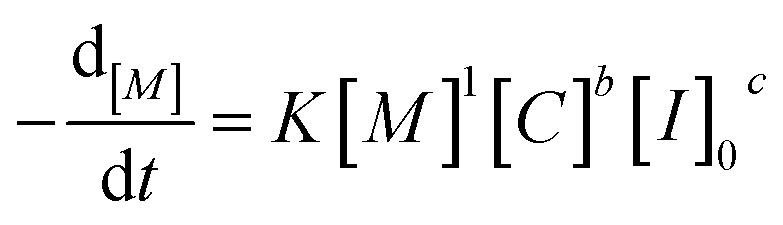
where [*M*] is the concentration of the monomer, [*C*] is the concentration of the catalyst, and [*I*]_0_ is the initial concentration of the initiator.

To determine the reaction order of the initiator (*c* in eqn (1)), the rate law equation was converted into the following equation:2
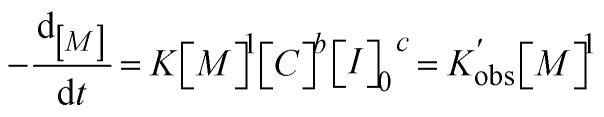
3
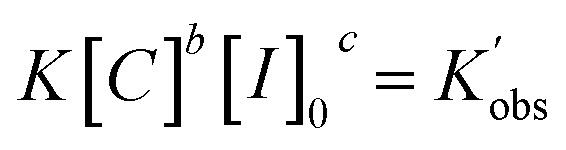


Taking the natural logarithms of both sides of eqn (4), the following relationship will be obtained:4
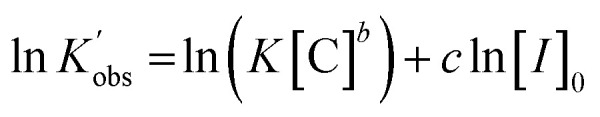


Next, the initiator dependency was explored using data from the sliced degree of polymerization experiments by plotting 
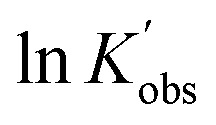
 against ln[*I*]_0_, while keeping the catalyst concentration constant (as shown in Fig. 3(B)). First-order fitting across initial monomer concentrations ranging from 0.2 to 0.7 mol L^−1^ was conducted and presented in Fig. S20. Under those flooding conditions, the rate of the polymerization exhibits a clear half-order dependency in initiator loading with an average value of 0.54. Fractional dependencies have been reported in the ROP of Lactide and lactone with zinc,^29,30^ aluminium,^47–49^ tin(ii),^50^ and N-heterocyclic^37^ related catalysts, resulting from aggregation of the active species in the polymerization medium. In the ROP of l-lactide catalyzed by TBD, the catalyst acts as a strong base, and the initiator is first deprotonated by TBD to generate the active alkoxide species (RO^−^) that is capable of initiating ring-opening. Analysis of the concentration of RO^−^ at various initial monomer concentrations and DPs reveals a clear linear correlation between [RO^−^] and the square root of the initial initiator concentration, [ROH]^0.5^, as shown in Fig. 3(E). The calculation details for estimating the partial deprotonation of the initiator by TBD are provided in the SI. This correlation supports the experimentally observed half-order dependence of the polymerization rate on the initial initiator concentration.

As for the reaction order of ROP of l-lactide in catalyst (TBD), the same derivation for initiator order determination was applied to eqn (3), resulting in the following equation:5
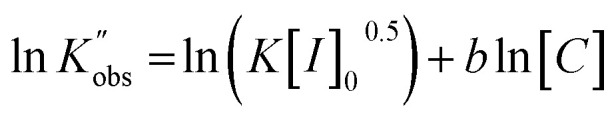
With the data sliced from monomer-to-catalyst ratio sweep experiments, Fig. 3(C) illustrates the fitting of 
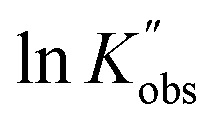
 against ln[*C*]. The fitting shows a good linearity, with the slope values being very close to one. As presented in Fig. 3(F), the average values (1.23) of the fitting across the initial monomer concentrations starting from 0.2 to 0.7 mol L^−1^ confirmed the first-order dependency of the catalyst on the rate law, resulting in the overall rate law of the polymerization shown below,6
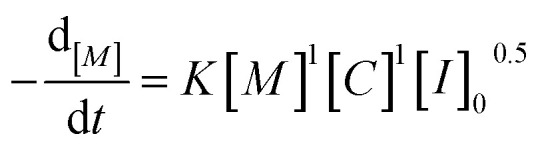


**Scheme 2:**
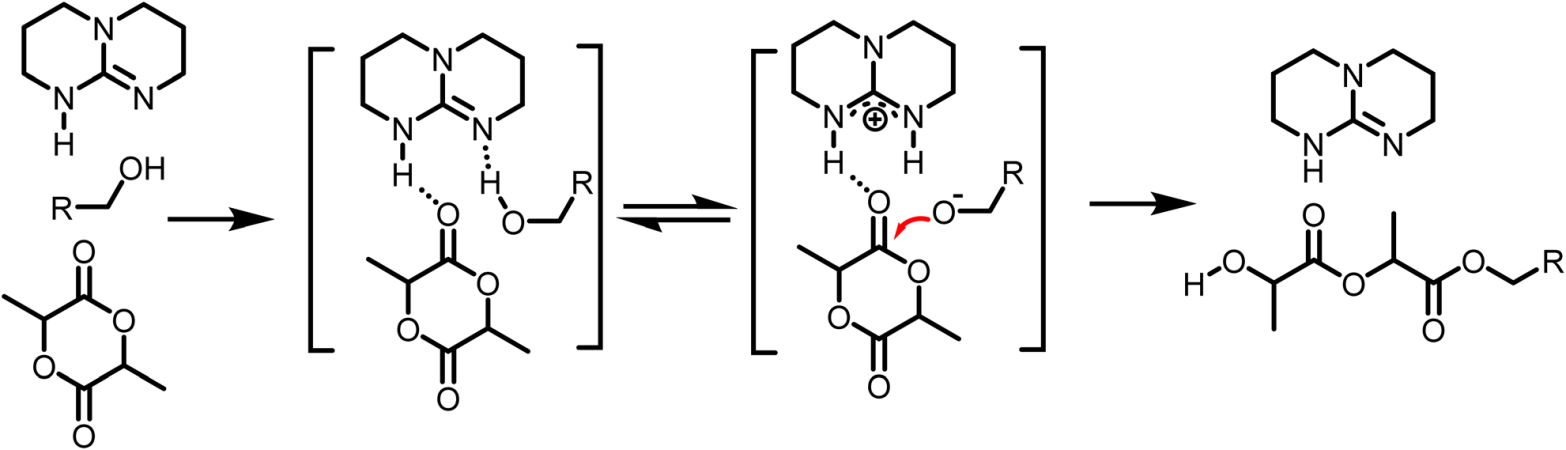
Proposed mechanism of TBD catalyzed ROP of l-lactide by hydrogen bonding from the determined reaction order.

Based on the final overall rate law, although a significantly faster organocatalyst (TBD) is employed in this study, the same first-order dependencies on monomer concentration as reported for zinc or stannous octoate catalyst systems are overall observed, underpinning their mechanistic similarities.^29,35,51^ Guided by the derived rate law, a mechanism for the TBD-catalyzed ring-opening polymerization of l-lactide, initiated by 4-methylbenzyl alcohol, is proposed and illustrated in Scheme 2. In the initial step, TBD, l-lactide, and 4-methylbenzyl alcohol form a hydrogen-bonded intermediate complex. TBD then partially and reversibly deprotonates the initiator to generate the active alkoxide species (RO^−^), which subsequently performs the rate-determining step, a nucleophilic attack on the carbonyl carbon of the l-lactide ring, resulting in the ring opening and chain propagation.

With the rate law of ROP of l-lactide catalyzed by TBD being established, the gate to a deeper understanding of the reaction was opened. As presented in Fig. 3(A), the ROP of l-lactide catalyzed by TBD is a first-order reaction in monomer, and consequently, linear first plots can be constructed from the obtained data. For each plot as given in Fig. S16, after converting to ln(*M*_0_*/M*_*t*_), 3-dimensional plots of ln(*M*_0_*/M*_*t*_) against residence time and monomer concentration were obtained (Fig. S22). Then, *k*_obs_ were fitted out from for all different monomer concentrations.

**Fig. 4:**
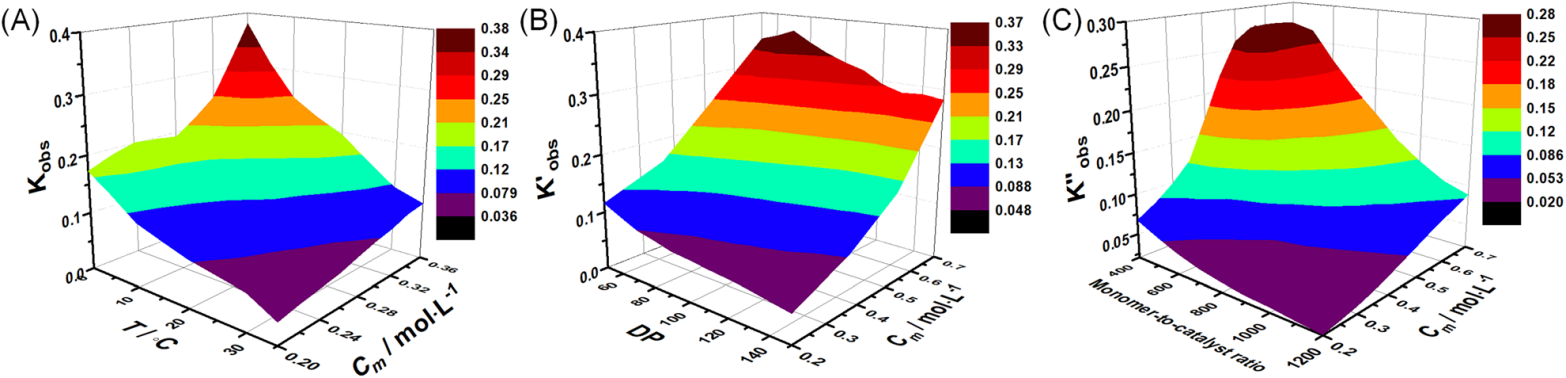
3-Dimensional surface plots of *K*_obs_ obtained from (A) concentration-sweeping experiments, (B) degree of polymerization sweeping experiments and (C) monomer-to-catalyst ratio sweeping experiments.

With these fits, it is possible to visualize the entire parameter space by plotting *k*_obs_ of different monomer concentrations at various temperatures. This allows for directly assessing the rate of polymerization and its behaviour. A view of *K*_obs_ increasing with increasing monomer concentration and decreasing temperature is presented in Fig. 4(A). Similarly, 3D profiles (Fig. 4(B) and (C)) of how *K*_obs_ are influenced by the degree of polymerization and monomer-to-catalyst ratio were generated from the degree of polymerization sweeping data (Tables S17–S20) and monomer-to-catalyst sweeping data (Tables S12–S15), respectively. These profiles allow us to investigate the activation energy of the ROP of l-lactide across the entire monomer concentration range studied in the following section. With known observed rate constants (*K*_obs_, 
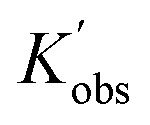
, and 
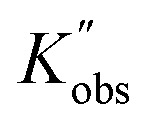
) from all experiments, the actual rate constants (*K*) of experiments carried out at 20 °C are calculated based on eqn (3), and presented in Fig. S23. As shown, after accounting for differences in initiator and catalyst concentrations, the corrected rate constants obtained from (i) monomer concentration sweeping experiments, (ii) degree of polymerization sweeping experiments, and (iii) monomer-to-catalyst ratio sweeping experiments carried out at 20 °C are 1883 ± 369, 1744 ± 368, and 2180 ± 470, respectively. These values are in good agreement with one another. Furthermore, the rate constant remains independent of the initial monomer concentration, confirming the first-order dependence on monomer. These profiles allow us to investigate the activation energy of the ROP of l-lactide across the entire monomer concentration range studied in the following section.

**Fig. 5:**
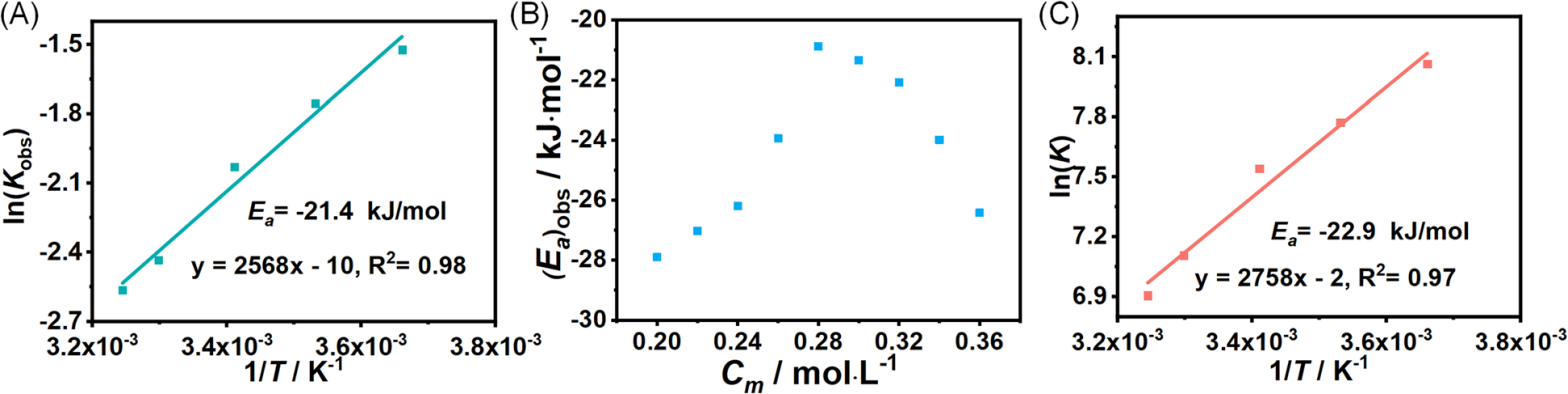
(A) Plots of ln(*k*_obs_) against temperature at initial monomer concentration varying from 0.2 to 0.36 M, (B) the summary of observed activation energy generated from the fitting plots and (C) plot of ln(*K*) against temperature.

Beyond the identification of reaction order and observed reaction rate, another important aspect of TBD catalyzed ROP of l-lactide that is still not addressed yet is (*E*_a_)_obs_, and the 3D plot of *K*_obs_ against monomer concentration and temperature (Fig. 4(A)) provides us with the tool to explore (*E*_a_)_obs_ in detail. ln(*K*_obs_) was plotted against 1/*T* to get the activation energy according to the Arrhenius equation, and an example of the plot with 0.3 M initial monomer concentration was provided in Fig. 5(A), giving an (*E*_a_)_obs_ of −21.4 kJ mol^−1^. Negative (*E*_a_)_obs_ were obtained from Arrhenius fits using initial monomer concentrations ranging from 0.2 to 0.36 mol L^−1^, as shown in Fig. 5(B). This observation explains the decrease in monomer conversion with increasing temperature. In contrast to the negative observed activation energy observed in this study, previously reported activation energies for the ring-opening polymerization (ROP) of l-lactide are predominantly positive, ranging from 58 to 86 kJ mol^−1^ across various systems.^32–35,52^ These positive values are characteristic of polymerizations catalyzed by metallic catalysts, which typically follow a coordination-insertion mechanism. This mechanism is governed by coordination chemistry, where the stability of metal–ligand bonds is relatively insensitive to temperature variations. Consequently, elevated temperatures increase the kinetic energy of reacting species, thereby facilitating the rate-determining monomer insertion step and resulting in a positive apparent activation energy. This discrepancy is an interesting observation given the high similarity that is identified for the reaction orders discussed above. Here, the ROP of l-lactide catalyzed by TBD can be seen as a two-step reaction; the formation of the intermediate is the first step, and the ring opening of l-lactide is the second step, where the formation of the intermediate highly relies on the hydrogen bonds, while the ring opening is driven by the exothermic enthalpic contribution from the release of ring strain. l-lactide, as a low-strain monomer, has been reported to have higher monomer equilibrium concentrations at higher reaction temperatures.^36,53,54^ This is consistent with the data shown in Fig. 2(A), which confirms that the monomer concentration at equilibrium increases as the temperature rises. Therefore, the increase in reaction temperature will decrease the reaction rates of intermediate formation and ring opening of l-lactide, thus leading to a decrease in the overall reaction rate.

## Conclusions

The ROP of l-lactide catalyzed by TBD was efficiently screened on a lower second time resolution on an optimized programmable robotic flow platform with excellent accuracy (absolute error of conversion <5%) and high reproducibility within the explored chemical space. Consistent conversion data in the studied reaction space were comprehensively revealed for the first time. With the massive data collected, the rate law of the ROP was developed, including a first-order dependency in l-lactide, a first-order dependency in TBD and a half-order dependency in 4-methylbenzyl alcohol. Such comprehensive kinetic data is available for a TBD catalyzed ROP here for the very first time, making the determinations much more robust compared to previous studies, where usually only a few datapoints were used for conclusions, rendering determinations statistically less meaningful. Additionally, detailed profiles of *K*_obs_ increase against the increase of monomer concentration, catalyst concentration and initiator concentration, and the decrease of reaction temperatures were presented. What's more, for the first time to our knowledge, a negative (*E*_a_)_obs_ was reported. The polymerization data library we developed allows users to identify optimal reaction conditions for their desired poly(l-lactide) synthesis. Additionally, the rate law we established within the studied chemical space provides access to the kinetic profiles of the ROP of l-lactide and any other monomer. This is critical for the synthesis of polyesters with the desired molecular weight and *Đ* for a specific application. Solvent polarity and hydrogen-bonding capabilities can influence both the rate constants and the activation energies, as they affect the stability of the intermediate complexes and the deprotonation process. Therefore, while the rate law is applicable within the conditions studied, extrapolating to higher concentrations or different solvents would require further experimental validation.

## Author contributions

Bo Zhang: conceptualization, methodology, visualization, investigation, validation, writing, original draft. Tanja Junkers: conceptualization, writing, reviewing and editing, supervision.

## Conflicts of interest

There are no conflicts to declare.

## Supplementary Material

SC-017-D5SC03998C-s001

## Data Availability

All raw data from the experiments is available online. The raw data are available in a Monash research repository at https://doi.org/10.26180/27824139.v2. All Python scripts used are available in a GitHub repository at https://github.com/PRDMonash/FTIR_screening_of_ROP_of_L-lactide. Supplementary information (SI) is available. See DOI: https://doi.org/10.1039/d5sc03998c.
