# Supplementary material for: Multidimensional kinetic study on the organocatalyzed ring-opening polymerization (ROP) of l-lactide via a robotic high-throughput flow platform
Source: Chem Sci. 2025 Dec 26;17(9):4706–14. doi: 10.1039/d5sc03998c (PMC12801520; doi:10.1039/d5sc03998c)
Supplement: SC-017-D5SC03998C-s001 [file SC-017-D5SC03998C-s001.pdf]

Supplementary Information (SI) for Chemical Science.
This journal is © The Royal Society of Chemistry 2026

Supporting Information for

Multidimensional Kinetic Study on the Organocatalyzed Ring-Opening Polymerization (ROP) of L-lactide via a Robotic High-Throughput Flow Platform

Bo Zhang, and Tanja Junkers*

Polymer Reaction Design group, School of Chemistry, Monash University, 19 Rainforest Walk, Building 23, Clayton, VIC 3800, Australia.

*Tanja Junkers; Email: Tanja.Junkers@monash.edu

Table of Contents

1. Experimental procedures ... 3
Materials ... 3
Automated Screening Flow Platform ... 3
Experimental ... 4
2. Measurements and analysis ... 8
Fourier transform infrared (FT-IR) ... 8
High-field nuclear magnetic resonance (NMR) ... 8
Size Exclusion Chromatography (SEC) ... 9
3. Supplementary results ... 9
Conversion data from FT-IR ... 9
Molecular weight distribution data from SEC ... 14
3-dimensional polymerization data ... 15
First-order linear plots for concentration-sweep experiments ... 31
First-order linear plots for degree of polymerization sweep experiments ... 32
First-order linear plots for monomer-to-catalyst ratio sweep experiments ... 33
Deprotonation of 4-methylbenzyl alcohol by TBD ... 34
3-dimensional first-order linear plots ... 35
Plots of the actual rate constant from different experiments ... 35
4. Raw data access ... 35

1. Experimental procedures

Materials

L-lactide (Hebei Guanlang Biotechnology Co., Ltd., 99%) was purified via passing through a silica column first and then recrystallized with toluene three times, then dried under high vacuum at room temperature for at least four days. 1,5,7-Triazabicyclo[4.4.0]dec-5-ene (Merck, 98%), also known as TBD was used as received. 4-Methylbenzyl alcohol (98%) was brought from Sigma-Aldrich recrystallized with petroleum ether twice, and dried under a vacuum. L-lactide, TBD and 4-methylbenzyl alcohol were all stored in the glovebox after being purified and dried. Benzoic acid was purchased from Chem-supply and used as received. HPLC-grade dichloromethane (DCM) was brought from Chem-supply and purified with a solvent purification system (SPS) before being used.

Automated Screening Flow Platform

The programmable high throughput flow platform was established via combining machine automation, flow chemistry and inline analysis, a scheme of the robotic platform is shown below (scheme S1). An inline quenching of benzoic acid solution was introduced to terminate the ring-opening polymerization (ROP) at a given residence time. Inline FT-IR was used to track the chemical change in the reaction solution in real-time. A Python script was running on the computer to control the flow rates of different streams based on users' preferences and perform the data analysis.

Table S1. Overview of the flow parts used in the platform

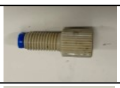	IDEX XP-230	Flangeless Fitting Natural, PEEK, 1/4- 28 Flat-Bottom, for 1/16" OD
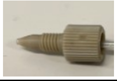	IDEX F-120	One-Piece Fingertight 10-32 Coned, for 1/16" OD Natural
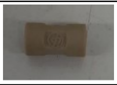	IDEX P-702	Union Assembly PEEK .020 thru hole, for 1/16" OD"
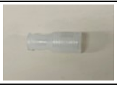	IDEX P-627	Threaded Adapter with Fitting, PEEK, 0.020" ID, 10-32 Coned (F) to 1/4-28 Flat Bottom (F)
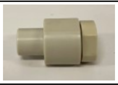	IDEX CV-3315	Inline Check Valve, Inlet, 3 psi, 0.02" ID, 1/4-28 (F) to 1/4-28 (M) Flat Bottom
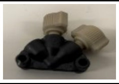	IDEX U-466	High-Pressure Static Mixing Tee Assembly, PEEK, 0.020" ID, 1/16" OD Tubing, 10-32 Coned, 10 µm UHMWPE Frit
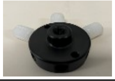	IDEX P-170	High-Pressure Manifold Assembly, 7 Port, PEEK, 0.020" ID, 1/16" OD Tubing, 10-32 Coned

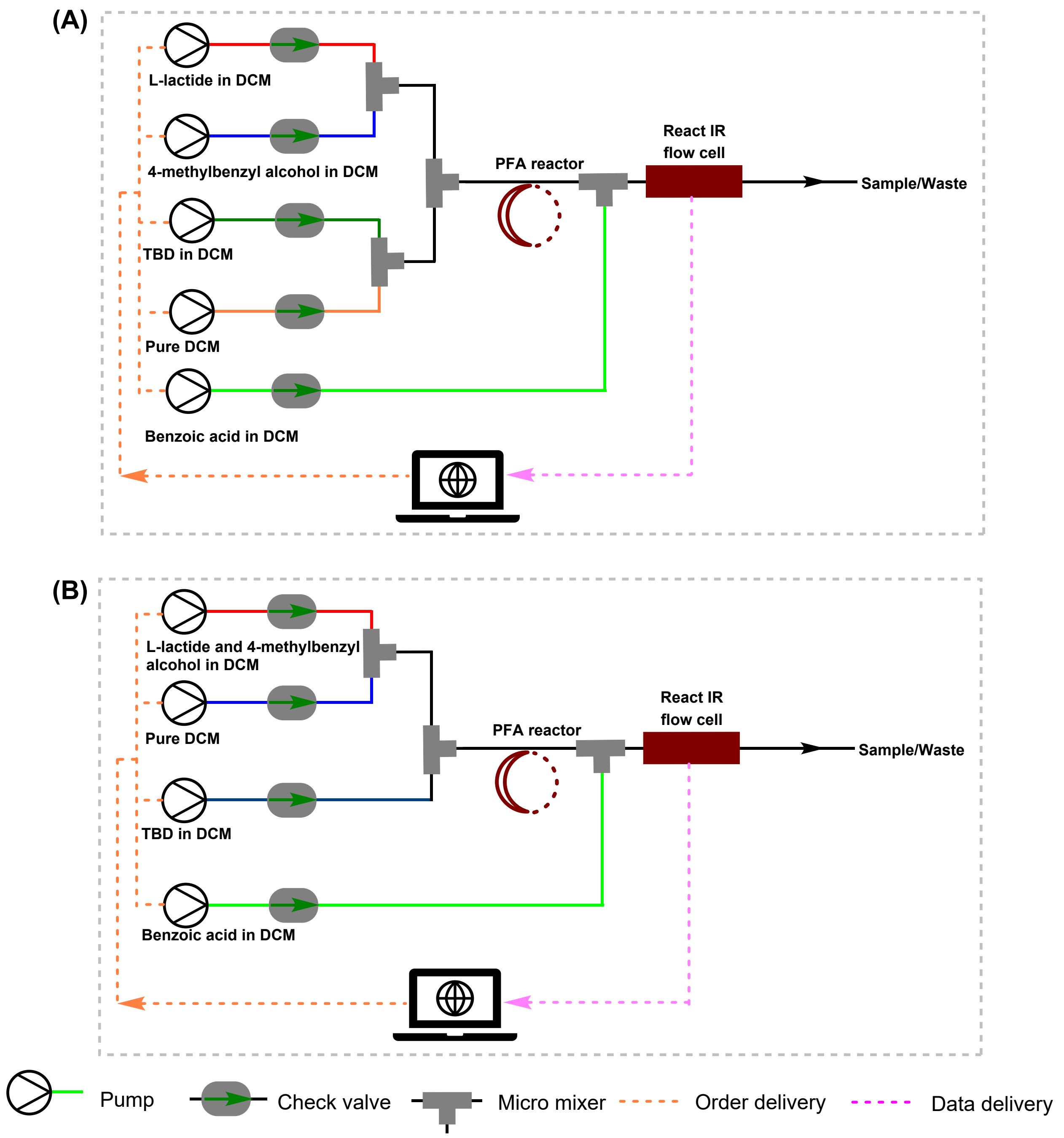

Scheme S1. Scheme of the programmable robotic platform for (A) degree of polymerization screening, and (B) monomer concentration screening and monomer-to-catalyst ratio screening.

Experimental

Separate stock solutions of monomer (L-lactide), initiator (4-methylbenzyl alcohol), and catalyst (TDB) were prepared with HPLC-grade DCM from the SPS system and transferred to 50 ml gastight syringes (Trajan Scientific) in the glove box. The stock solution of quenching agent (4-methylbenzoic acid) was prepared with normal DCM. Dry DCM was loaded first to wash the whole system for at least 3 minutes. Then, all the stock solutions were loaded onto the system. Syringe pumps from Chemyx were used to deliver stock solutions of monomer, catalyst and initiator, while the quenching agent was delivered by a peristaltic pump. With pre-programmed Python script, pumps delivered the stock solution with desired flowrates using PFA tubing (0.75 mm I.D., 1/16’’ O.D.), after mixing with micromixers, the mixed reagents were passed to the reactor (0.01678 ml, 0.08333 ml, 0.1678 ml) placed in an ice bath (0 ˚C, 10 ˚C, 20 ˚C, 30 ˚C, 35 ˚C).

Screening monomer concentration and residence time of L-lactide ROP polymerization with the robotic platform (Monomer concentration sweeping experiments)

In a typical procedure, three stock solutions were prepared in volumetric flasks. One stock solution containing L-lactide and 4-methylbenzyl alcohol (1 M and 0.01 M, respectively) and one containing TBD (0.03M) were prepared with dry DCM in the glovebox, along with pure dry DCM, they were transferred to three 50 ml gastight syringes, separately. A stock solution of 4-methylbenzoic acid (1 M) was prepared in the fume hood. Dry DCM was first used to wash the robotic platform for at least 3 minutes, and then stock solutions were loaded onto the platform. The concentration of stock solutions, reactor parameters (volume of reactor, volume of tubing between reactor and the FT-IR chamber) and reaction parameters (the equivalent of monomer to initiator and catalyst is 100:1:0.5) were set manually beforehand. After starting the Python script, orders will be given to different pumps to deliver different streams with desired flow rates (as shown in Figure S2). Calculations of the flow rates are as follows:

$$Flow_{total} = \frac{V_{reactor}}{t_{res}}$$ Equation (S1)

$$Flow_{M} = \frac{C_{M,D} * Flow_{total}}{C_{M,S}}$$ Equation (S2)

$$Flow_{Cat} = \frac{C_{M,D} * Flow_{total}}{R_{MCR} * C_{Cat,S}}$$ Equation (S3)

$$Flow_{S} = Flow_{total} - Flow_{M} - Flow_{Cat}$$ Equation (S4)

In which, $V_{reactor}$ is the volume of the reactor, mL; t_{res} is the desired residence time, second; $Flow_{total}$ is the total flow rate, mL/min; $Flow_{M}$ is the flow rate of monomer, mL/min; $Flow_{Cat}$ is the flow rate of the catalyst, mL/min; $Flow_{S}$ is the flow rate of solvent (Pure DCM), mL/min; R_{MCR} is monomer to catalyst ratio; $C_{M,S}$ is the concentration of monomer stock solution, mol/L; $C_{M,D}$ is the desired monomer concentration, mol/L ; $C_{Cat,S}$ is the concentration of the catalyst stock solution.

After mixing, reaction mixtures with monomer concentrations ramping from 0.2 M to 0.8 M will be delivered to the reactor, and the quenching agent will terminate the reaction right after it comes from the reactor and the chemical transition will be characterized by the inline FT-IR placed at the end of the platform. The reaction was performed at 0 ˚C, 10 ˚C, 20 ˚C, 30 ˚C and 35 ˚C with different residence times (1, 5 and 10 seconds). For all the concentration-sweeping experiments, parallel experiments were carried out to validate the reproducibility of the reaction on the platform. Samples were collected at the end of all experiments for offline high-field NMR and SEC analysis.

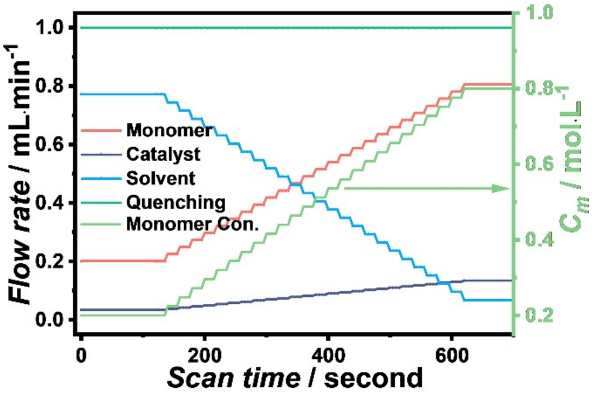

Figure S1. Flow rates of different streams for monomer concentration-sweep experiments.

Screening monomer to catalyst ratio and residence time of L-lactide ROP polymerization with the robotic platform (Monomer to catalyst ratio sweeping experiments)

In a typical procedure, three stock solutions were prepared in volumetric flasks. One stock solution containing L-lactide and 4-methylbenzyl alcohol (1 M and 0.01 M, respectively) and one containing TBD (0.03M) were prepared with dry DCM in the glovebox, along with pure dry DCM, they were transferred to three 50 ml gastight syringes, separately. A stock solution of 4-methylbenzoic acid (1 M) was prepared in the fume hood. Dry DCM was first used to wash the robotic platform for at least 3 minutes, and then stock solutions were loaded onto the platform. The concentration of stock solutions, reactor parameters (volume of reactor, volume of tubing between the reactor and the FT-IR chamber) and reaction parameters (the equivalent of monomer to initiator is 100:1) were set manually beforehand. After starting the Python script, orders will be given to different pumps to deliver different streams with desired flow rates (as shown in Figure S3, the calculation of the flow rates is the same as that of the monomer concentration sweeping experiments), after mixing, reaction mixtures with monomer-to-catalyst ratio ramping from 200 to 1200 will be delivered to the reactor, and the quenching agent will terminate the reaction right after it comes from the reactor and the chemical transition will be characterized by the inline FT-IR placed at the end of the platform. The reaction was performed at 20 ˚C with different initial monomer concentrations (0.2 M, 0.35 M, 0.5 M and 0.7 M) and residence times (1, 5 and 10 seconds). Parallel experiments were carried out to validate the reproducibility of the reaction on the platform for all the monomer-to-catalyst ratio sweeping experiments. Samples were collected at the end of all experiments for offline high-field NMR and SEC analysis.

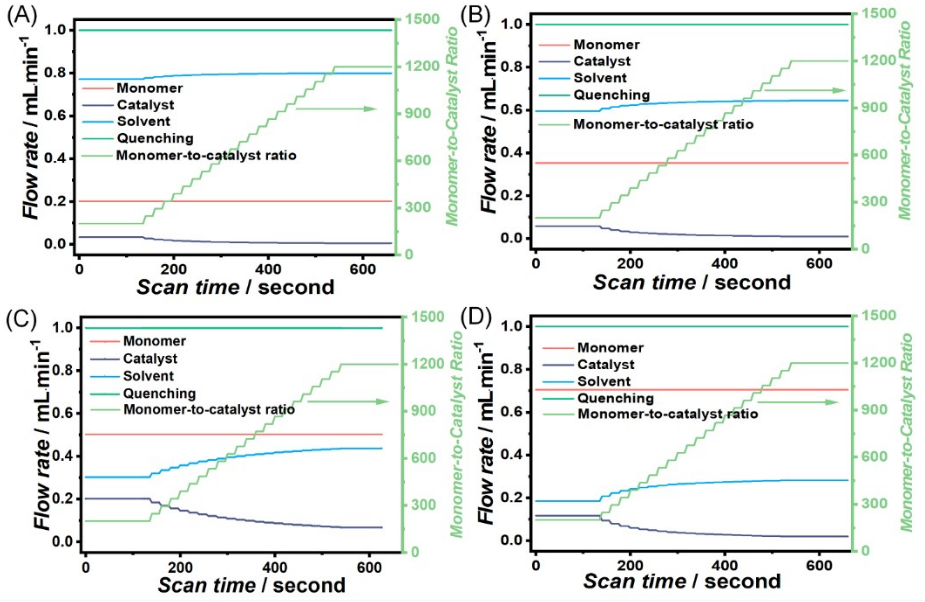

Figure S2. Flow rates of different streams for monomer-to-catalyst ratio sweep experiments with different initial monomer concentrations: (A) 0.2 M, (B) 0.35M, (C) 0.5 M and (D) 0.7 M.

Screening degree of polymerization (DP) and residence time of L-lactide ROP polymerization with the robotic platform (DP sweeping experiments)

In a typical procedure, four stock solutions were prepared in volumetric flasks. One stock solution containing L-lactide (1 M), one consisting 4-methylbenzyl alcohol (0.1 M) and one containing TBD (0.03M) were prepared with dry DCM in the glovebox, along with pure dry DCM, they were transferred to three 50 ml gastight syringes, separately. A stock solution of 4-methylbenzoic acid (1 M) was prepared in the fume hood. Dry DCM was first used to wash the robotic platform for at least 3 minutes, and then stock solutions were loaded onto the platform. The concentration of stock solutions, reactor parameters (volume of reactor, volume of tubing between reactor and the FT-IR chamber) and reaction parameters (the equivalent of monomer to catalyst is 300:1) were set manually beforehand. After starting the Python script, orders will be given to different pumps

to deliver different streams with desired flow rates (as shown in Figure S4). Flow rates of different streams were calculated as follows:

$$Flow_{total} = \frac{V_{reactor}}{t_{res}}$$ Equation (S5)

$$Flow_M = \frac{C_{M,D} * Flow_{total}}{C_{M,S}}$$ Equation (S6)

$$Flow_{Cat} = \frac{C_{M,D} * Flow_{total}}{R_{MCR} * C_{Cat,S}}$$ Equation (S7)

$$Flow_{Ini} = \frac{C_{M,D} * Flow_{total}}{DP * C_{ini,S}}$$ Equation (S8)

$$Flow_S = Flow_{total} - Flow_M - Flow_{Ini} - Flow_{Cat}$$ Equation (S9)

In which, $V_{reactor}$ is the volume of the reactor, mL; t_{res} is the desired residence time, second; $Flow_{total}$ is the total flow rate, mL/min; $Flow_M$ is the flow rate of monomer, mL/min; $Flow_{Cat}$ is the flow rate of the catalyst, mL/min; $Flow_{Ini}$ is the flow rate of the initiator, mL/min; $Flow_S$ is the flow rate of solvent (Pure DCM), mL/min; R_{MCR} is monomer to catalyst ratio; DP is the degree of polymerization; $C_{M,S}$ is the concentration of monomer stock solution, mol/L; $C_{M,D}$ is the desired monomer concentration, mol/L; $C_{Cat,S}$ is the concentration of the catalyst stock solution, mol/L; $C_{Ini,S}$ is the concentration of initiator stock solution, mol/L.

After mixing, reaction mixtures with a theoretical DP at 100 % conversion ramping from 50 to 150 will be delivered to the reactor, and the quenching agent will terminate the reaction right after it comes from the reactor, and the chemical transition will be characterized by the inline FT-IR placed at the end of the platform. The reaction was performed at 20 ˚C with different initial monomer concentrations (0.2 M, 0.35 M, 0.5 M and 0.7 M) and residence times (1, 5 and 10 seconds). Parallel experiments were carried out for all the DP sweeping experiments to validate the reproducibility of the reaction on the platform. Samples were collected at the end of all experiments for offline high-field NMR and SEC analysis.

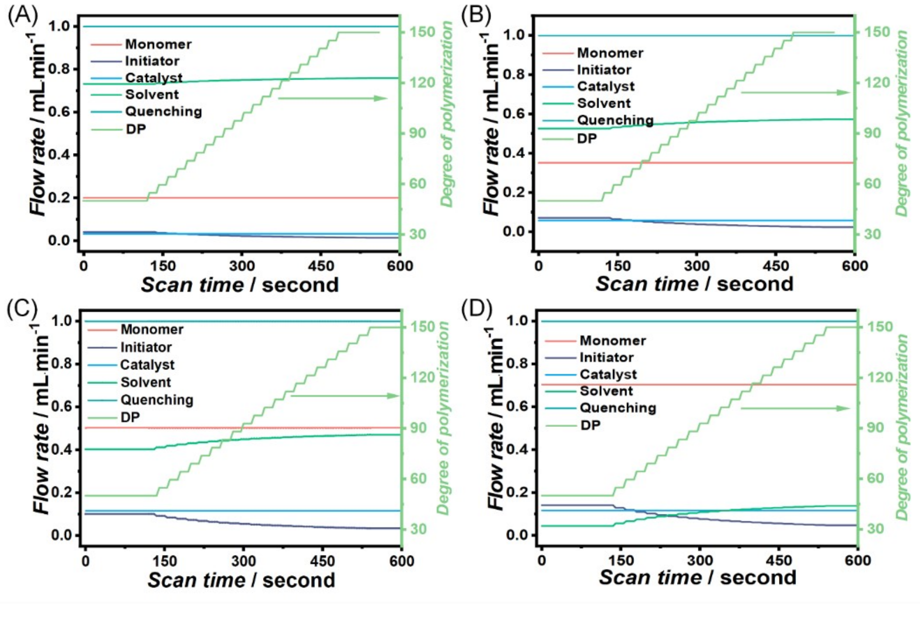

Figure S3. Flow rates of different streams for the degree of polymerization sweep experiments with different initial monomer concentrations: (A) 0.2 M, (B) 0.35M, (C) 0.5 M and (D) 0.7 M.

2. Measurements and analysis

Fourier transform infrared (FT-IR)

ReactIR (Mettler Toledo) with a 50 µL DS micro flow cell was used to real-time inline monitor the concentration of monomer. The equipment was operated by ICIR software, and the sample interval was 5 seconds. After finishing every sample, it will automatically generate a CSV file containing the raw data of the IR spectrum.

For the characterization of monomer conversion for the ROP of L-lactide, the ring breathing mode peak of L-lactide from 940 to 928 cm^{-1} was followed to calculate the concentration of L-lactide in the solution.[1] Firstly, a series of L-lactide samples with known concentrations (0.1M, 0.2 M, 0.3 M, 0.4 M and 0.5 M) was pumped through the microflow cell of FT-IR, and then the integrated peak area of the ring breathing was calculated via subtracting the peak area of background (mixture of 1 mL/min of pure DCM and 1 mL/min of quenching agent) in the range of 940 to 924 cm^{-1} from that of the known concentration samples. IR spectra of known concentration samples ranging from 940 to 924 cm^{-1} were provided in Figure S5 (A). With the concentration and peak area obtained, a nice linear fitting with an R-squared value of 0.999 was built between them (Figure S5 (B)). Then, the linear fitting was inserted into the Python script to analyse the concentration of L-lactide and give monomer conversion based on the initial concentration. IR spectra of the monomer-to-catalyst ratio sweeping experiment with 0.7 M initial L-lactide were presented in Figure S5 (C) as an example to show the high resolution of FT-IR.

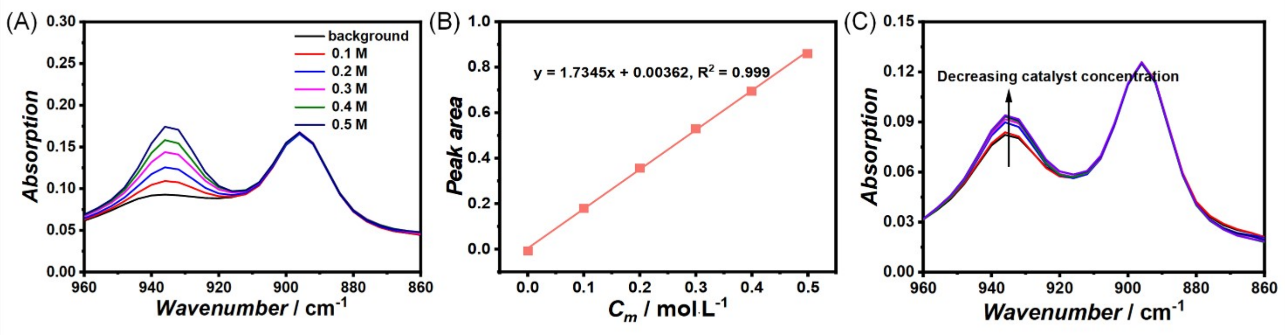

Figure S4. (A) IR spectra of L-lactide with concentrations ranging from 0 to 0.5 M in the range of 960 – 860 cm^{-1}, (B) linear fitting between the peak area of L-lactide ring breathing in the range of 940 – 924 cm^{-1} and L-lactide concentrations, and (C) IR spectra of the monomer-to-catalyst ratio sweeping experiment with 0.7 M initial L-lactide.

High-field nuclear magnetic resonance (NMR)

Monomer conversions of ROP were checked on a Bruker Avance III 400 MHz NMR spectrometer. The polymer samples were dissolved in deuterated chloroform after evaporating the solvent or purified with cold methanol. The conversion of the ROP was determined by integrating the CH group in the monomer (5.05 ppm) and the CH group in the polymer backbone (5.15 ppm).[2, 3]

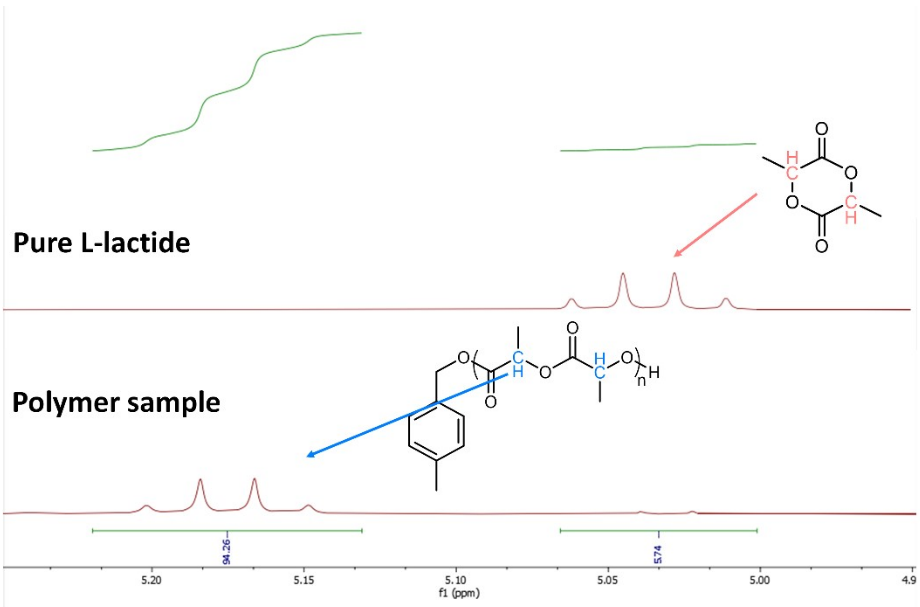

Figure S5. H^1-NMR spectra of Pure L-lactide and a sample of ROP of L-lactide in the range of 4.95 to 5.25 ppm.

Size Exclusion Chromatography (SEC)

The molecular weight and molecular weight distribution of the samples were analyzed on a PSS SECcurity2 GPC system, operated by PSS WinGPC software. The system is equipped with an SDV 5.0 µm guard column (50 x 8 mm), followed by three SDV analytical 5.0 µm columns with varying porosity (1000 Å, 100000 Å and 1000000 Å) (50 x 8 mm) and a differential refractive index detector. THF was used as the eluent at 40 ˚C with a 1 mL·min^{-1} flow rate. The GPC system was calibrated using linear narrow polystyrene standards from PSS Laboratories, ranging from 682 to 2.52 × 10^6 g·mol^{-1} PS (K = 14.1 x 10-5 dL·g^{-1} and α = 0.70).

3. Supplementary results

Conversion data from FT-IR

Conversion data for the concentration-sweep experiment

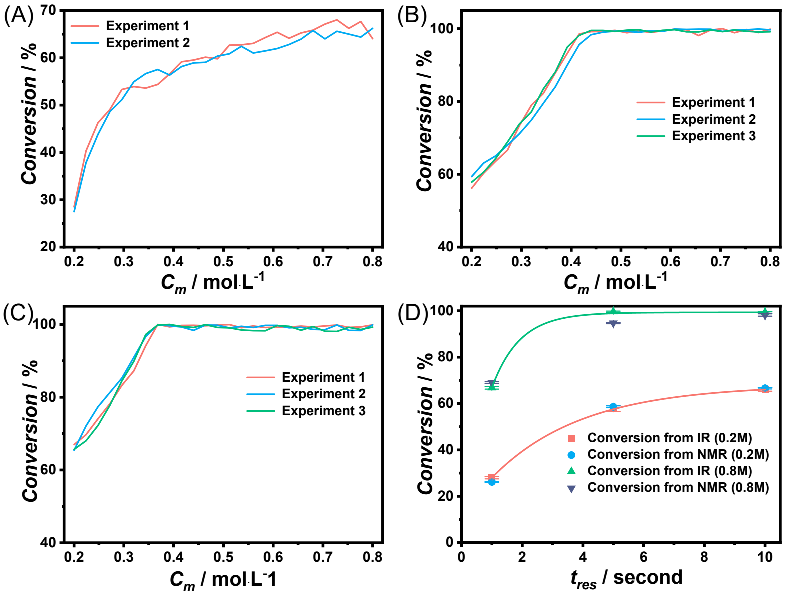

Figure S6. Conversion data of the concentration-sweep experiments carried out at 0 ˚C with (A) 1 second, (B) 5 seconds and (C) 10 seconds residence time, respectively, and (D) the summary of conversion at different residence times and different initial monomer concentrations.

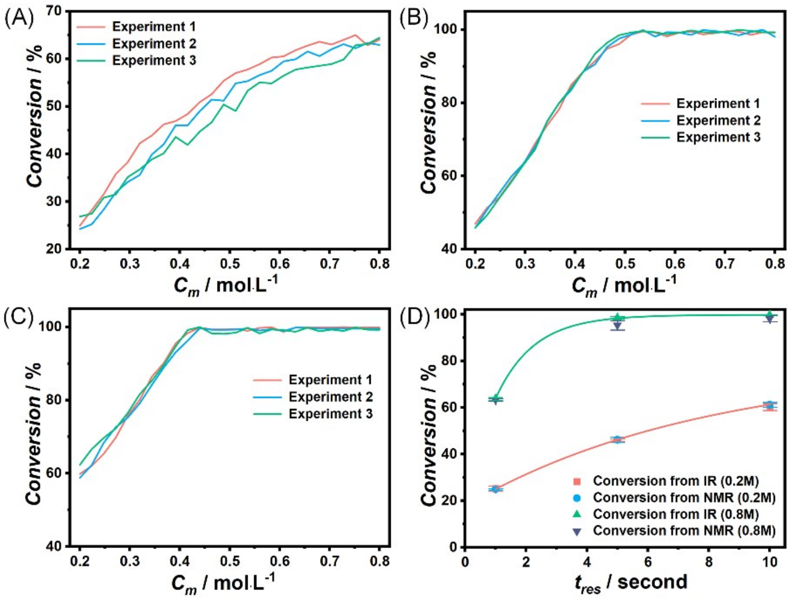

Figure S7. Conversion data of the concentration-sweep experiments carried out at 10 ˚C with (A) 1 second, (B) 5 seconds and (C) 10 seconds residence time, respectively, and (D) the summary of conversion at different residence times and different initial monomer concentrations.

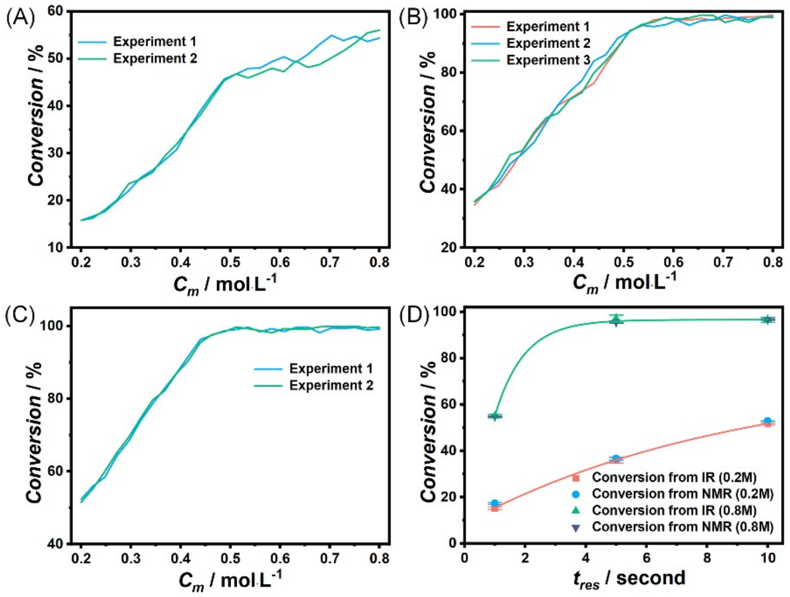

Figure S8. Conversion data of the concentration-sweep experiments carried out at 20 ˚C with (A) 1 second, (B) 5 seconds and (C) 10 seconds residence time, respectively, and (D) the summary of conversion at different residence times and different initial monomer concentrations.

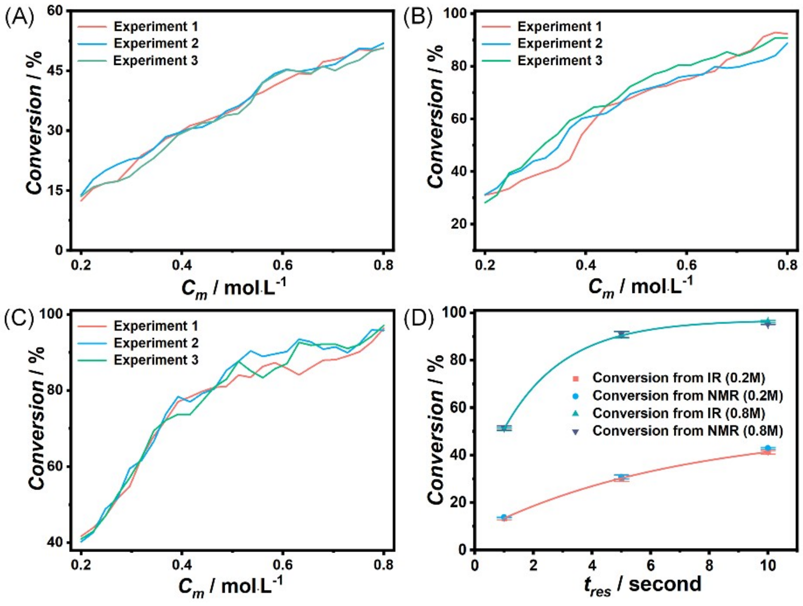

Figure S9. Conversion data of the concentration-sweep experiments carried out at 30 ˚C with (A) 1 second, (B) 5 seconds and (C) 10 seconds residence time, respectively, and (D) the summary of conversion at different residence times and different initial monomer concentrations.

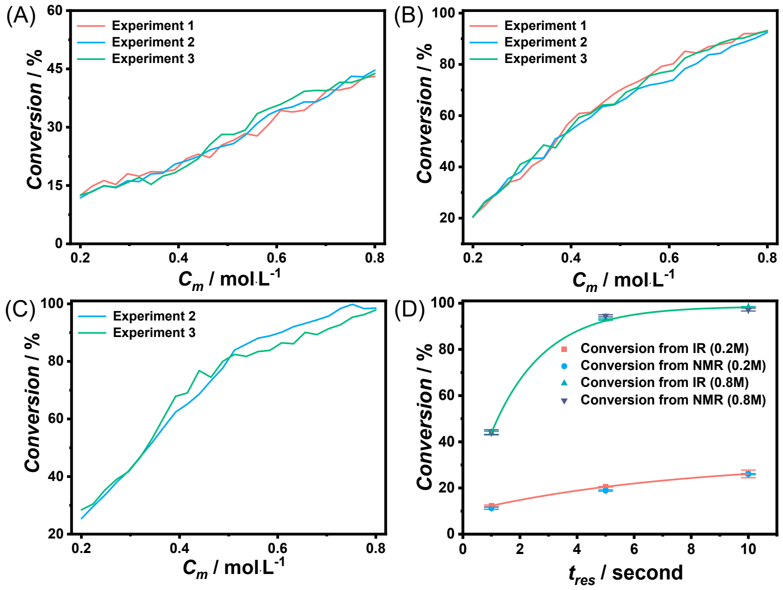

Figure S10. Conversion data of the concentration-sweep experiments carried out at 35 ˚C with (A) 1 second, (B) 5 seconds and (C) 10 seconds residence time, respectively, and (D) the summary of conversion at different residence times and different initial monomer concentrations.

Table S2. Summary of monomer conversion for concentration-sweeping experiments, and both NMR and IR conversions were the average result of the repeat experiments.

T / ˚C	t_{res} / sec	Initial C_m/ (mol·L^{-1})	*NMR Con. / %*	*IR Con. / %*	M_n/ kDa	*Đ*
0	1	0.2	26.2	28.0	6.2	1.26
		0.8	69.0	66.8	11.8	1.40
	5	0.2	58.6	57.8	9.8	1.29
		0.8	94.7	99.6	16.4	1.36
	10	0.2	66.6	66.0	10.0	1.26
		0.8	98.2	99.3	17.8	1.35
10	1	0.2	24.9	25.3	5.9	1.37
		0.8	63.1	63.8	10.1	1.37
	5	0.2	46.2	46.1	8.6	1.26
		0.8	95.3	98.5	18.5	1.28
	10	0.2	61.2	60.3	8.9	1.24
		0.8	98.2	99.7	18.3	1.35
20	1	0.2	18.3	15.3	6.6	1.39
		0.8	55.0	54.9	12.2	1.30
	5	0.2	36.7	35.4	7.3	1.18
		0.8	95.6	99.2	16.9	1.28
	10	0.2	52.9	51.7	8.7	1.22
		0.8	96.7	99.4	19.3	1.29
30	1	0.2	13.8	13.2	4.2	1.19
		0.8	51.5	51.0	10.0	1.36
	5	0.2	30.8	30.1	6.9	1.21
		0.8	91.0	90.6	13.6	1.29
	10	0.2	42.9	41.0	6.4	1.32
		0.8	95.1	96.4	18.2	1.28
35	1	0.2	11.2	12.3	3	1.11
		0.8	44.1	43.9	10.2	1.32
	5	0.2	18.9	20.5	4.8	1.15
		0.8	93.5	92.9	12.6	1.29
	10	0.2	26.0	26.1	4.8	1.21
		0.8	97.3	98.2	16.8	1.30

Conversion data for the monomer-to-catalyst ratio sweep experiment

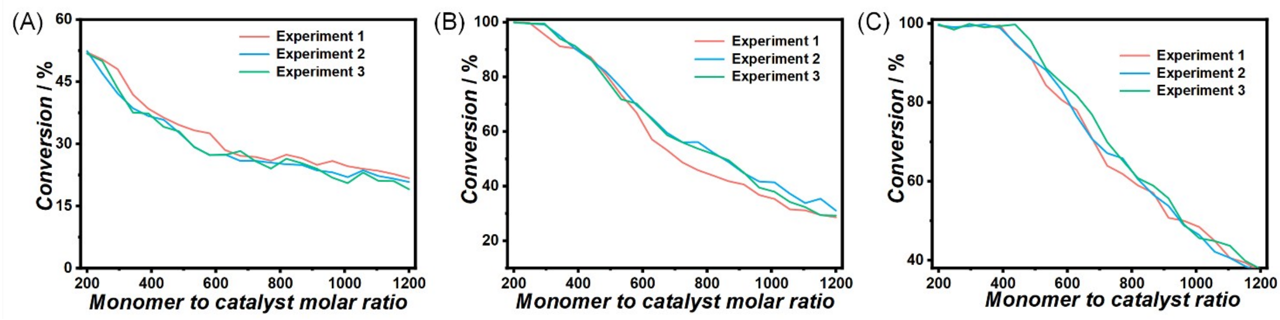

Figure S11. Conversion data of the monomer-to-catalyst ratio sweep experiments carried out at 20 ˚C with 0.5 M initial monomer concentration at (A) 1 second, (B) 5 seconds and (C) 10 seconds residence time, respectively.

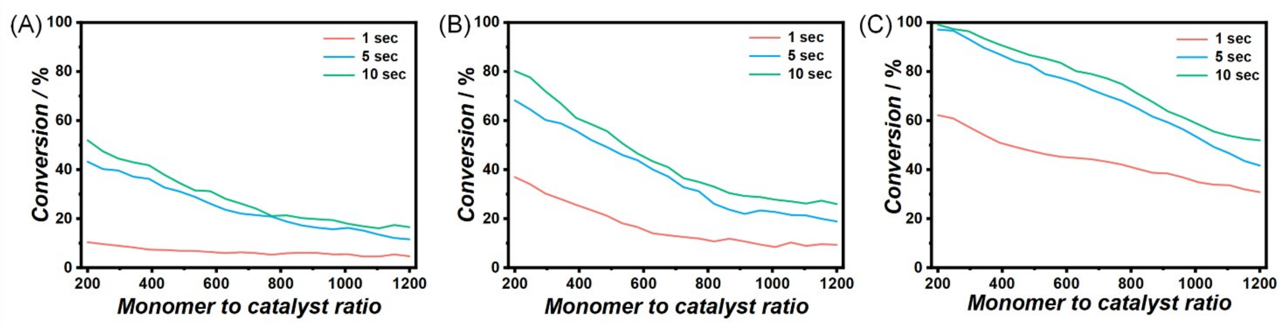

Figure S12. Conversion data of the monomer-to-catalyst ratio sweep experiments carried out at 20 ˚C with (A) 0.2 M, (B) 0.35 M and (C) 0.7 M initial monomer concentration, respectively.

Table S3. Summary of monomer conversion for monomer-to-catalyst ratio sweep experiments, and both NMR and IR conversions were the average result of the repeat experiments.

Initial C_m / (mol·L^{-1})	*t_{res}/ sec*	*Monomer-to-catalyst ratio*	*NMR Con. / %*	*IR Con. / %*

0.2	1	200	8.8	10.4
		1200	3.6	4.7
	5	200	40.8	43.2
		1200	10.3	11.6
	10	200	50.4	52.0
		1200	15.3	16.5
0.35	1	200	36.2	36.9
		1200	8.0	9.3
	5	200	66.4	68.2
		1200	17.3	18.9
	10	200	81.6	80.2
		1200	23.4	25.9
0.5	1	200	51.0	52.1
		1200	19.9	20.5
	5	200	96.5	99.9
		1200	28.6	29.6
	10	200	97.7	99.6
		1200	36.6	37.4
0.7	1	200	96.7	97.1
		1200	29.4	30.8
	5	200	96.7	97.1
		1200	39.5	41.6
	10	200	97.3	99.1
		1200	50.3	51.9

Conversion data for the degree of polymerization sweep experiment

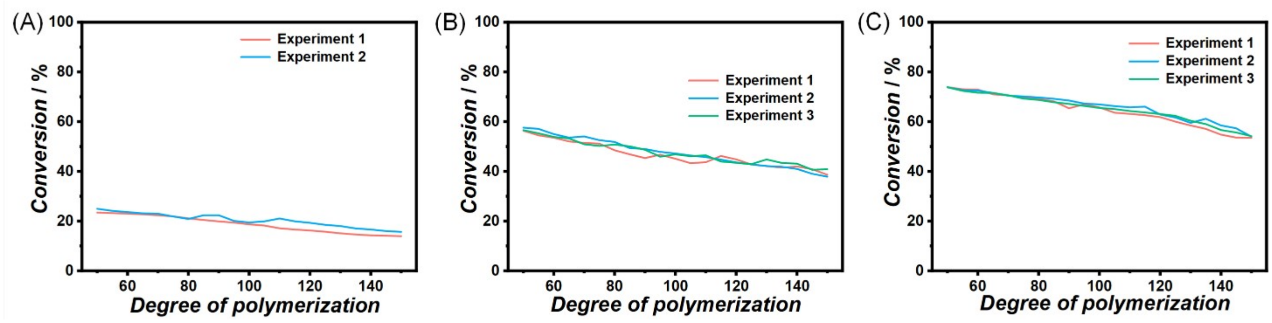

Figure S13. Conversion data of the degree of polymerization sweep experiments carried out at 20 ˚C with 0.35 M initial monomer concentration at (A) 1 second, (B) 5 seconds and (C) 10 seconds residence time, respectively.

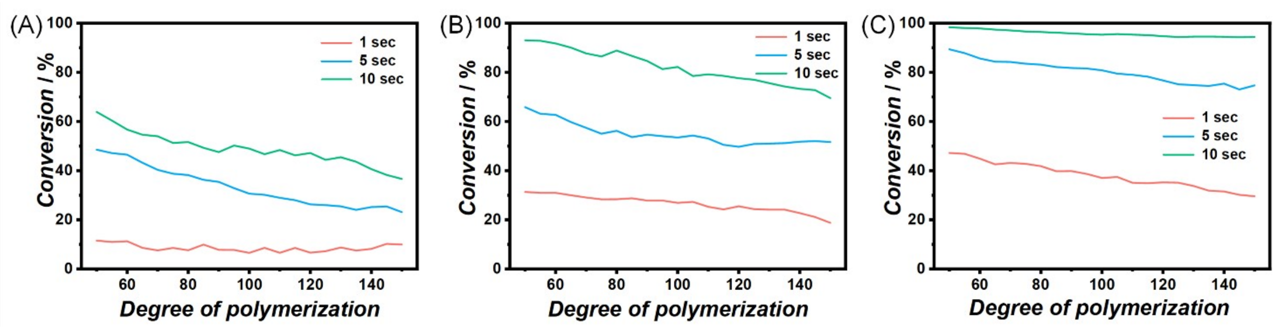

Figure S14. Conversion data of the degree of polymerization sweep experiments carried out at 20 ˚C with (A) 0.2 M, (B) 0.5 M and (C) 0.7 M initial monomer concentrations, respectively.

Table S4. Summary of monomer conversion for monomer-to-catalyst ratio sweep experiments, and both NMR and IR conversions were the average result of the repeat experiments.

Initial C_m/ (mol·L^{-1})	t_{res} / sec	*Degree of polymerization*	*NMR Con. / %*	*IR Con. / %*
0.2	1	50	9.9	11.5
		150	8.5	9.9
	5	50	46.7	48.5
		150	20.4	23.1
	10	50	65.9	63.9
		150	37.7	36.6
0.35	1	50	22.2	24.15
		150	14.7	14.8
	5	50	55.2	56.9
		150	38.6	38.8
	10	50	72.5	73.8
		150	51.6	53.8
0.5	1	50	29.6	31.4
		150	16.7	18.8
	5	50	67.7	65.8
		150	54.3	51.7
	10	50	94.2	93.0
		150	71.0	69.5
0.7	1	50	44.6	47.2
		150	29.3	29.6
	5	50	90.9	89.3
		150	77.8	74.7
	10	50	98.2	99.5
		150	95.4	94.4

Molecular weight distribution data from SEC

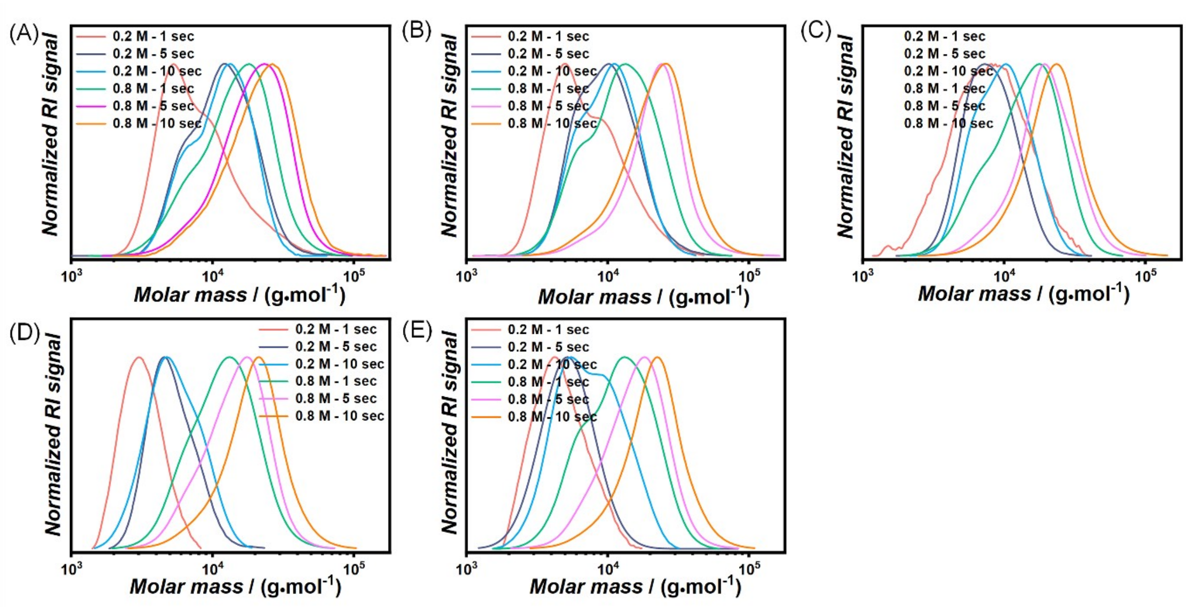

Figure S15. Polymer molecular weight distribution obtained from SEC for the concentration-sweep experiment at (A) 0 ˚C, (B) 10 ˚C, (C) 20 ˚C, (D) 30 ˚C and (E) 35 ˚C.

3-dimensional polymerization data

After collecting all the data generated from IR for each sweep experiment, a 3-dimensional plot was made with the Origin software, and a Python script was applied to fit the 3-dimensional plot with a 3rd-degree polynomial fitting. The fitting equation was as follows:

$$Z = A * X^3 + B * Y^3 + C * XY^2 + D * YX^2 + E * X^2 + F * Y^2 + G * XY + H * X + I * Y + J \qquad \text{Equation (S10)}$$

Z is monomer conversion (%), X is monomer concentration (M) or monomer-to-catalyst ratio or degree of polymerization, and Y is the residence time (1≤Y≤10). 3-dimensional plots of the sweeping experiments are listed in the following section.

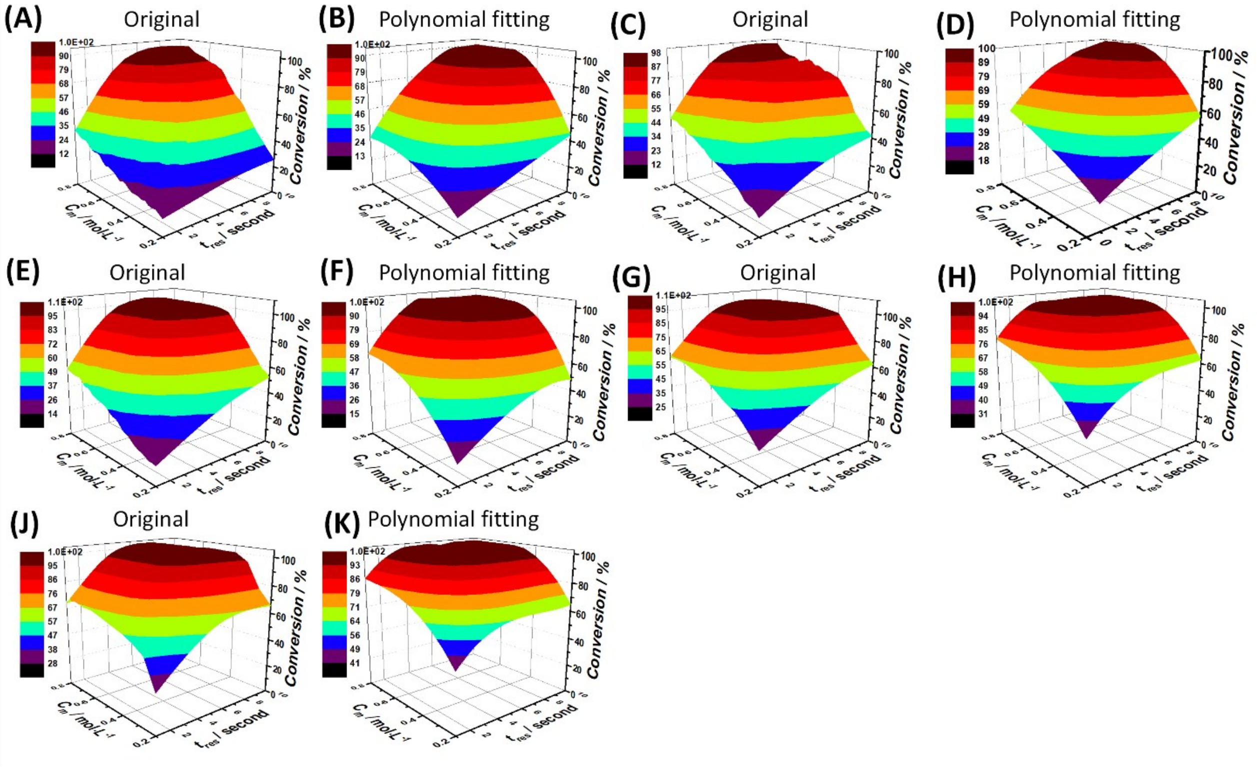

Figure S16. 3-dimensional surface plots of experimental data from concentration sweep experiments conducted at (A) 35 °C, (C) 30 °C, (E) 20 °C, (G) 10 °C, and (J) 0 °C; and corresponding three-dimensional surface plots from polynomial fitting at (B) 35 °C, (D) 30 °C, (F) 20 °C, (H) 10 °C, and (K) 0 °C.

Table S5. 3-dimensional fitting results of concentration-sweep data.

T/°C	*A*	*B*	*C*	*D*	*E*	*F*	*G*	*H*	*I*	*J*	$R^{2\,a}$	$R^{2\,b}$
35	35.308	-0.001	-1.640	-25.267	-105.771	0.314	51.077	72.371	-5.375	-4.964	0.998	0.997
30	26.178	0.009	-0.950	-25.355	-22.213	-0.064	37.405	40.227	-0.267	-0.578	0.990	0.989
20	66.497	-0.030	-1.770	-25.712	-202.756	0.438	45.089	196.471	-0.946	-30.947	0.992	0.986
10	171.571	0.068	-0.627	-15.581	-347.681	-1.808	20.398	268.770	15.170	-39.967	0.984	0.987
0	35.360	0.084	-0.405	-14.400	-157.565	-2.115	17.936	183.749	15.587	-17.475	0.983	0.980

a: is the R^2 value of fitting between the 3-dimensional polynomial fitting plots and the original 3-dimensional surface plot; b: is the R^2 value between the experimental data (shown in Figures S6 to S10) and the polynomial fitting.

Table S6. Conversion data from the concentration sweep experiment conducted at 35 °C, extracted from the original 3D surface plot.

Initial monomer concentration / M	*Conversion at different residence times (second) / %*									
	1	2	3	4	5	6	7	8	9	10
0.20	12.47	14.42	16.38	18.36	20.36	22.13	23.46	24.40	25.02	25.40
0.22	14.42	17.17	20.16	22.97	25.33	27.06	28.11	28.67	28.89	28.94
0.24	16.16	19.53	22.79	25.86	28.44	30.29	31.38	31.96	32.17	32.20
0.26	15.56	19.63	23.71	27.57	30.77	33.11	34.64	35.57	35.98	35.82
0.28	16.02	21.46	26.86	31.62	35.96	38.80	40.59	41.18	40.38	39.32
0.30	18.13	24.58	31.07	36.98	41.53	44.20	45.15	44.85	43.83	42.64
0.32	17.39	24.93	32.07	38.33	43.23	46.41	48.03	48.43	47.93	46.85
0.34	18.37	26.72	35.08	42.47	48.06	51.46	52.69	52.61	51.82	50.95
0.36	18.62	27.43	35.73	42.33	47.64	50.98	53.15	54.56	55.18	55.29
0.38	18.46	27.35	35.55	43.31	49.86	54.59	57.93	59.51	60.06	60.00
0.40	19.88	30.00	39.97	48.97	56.21	60.98	63.64	64.63	64.31	63.50
0.42	22.33	33.20	43.56	52.66	59.65	64.16	66.42	67.02	66.55	65.62
0.44	23.06	33.98	44.37	53.55	60.86	65.72	68.39	69.40	69.29	68.60
0.46	22.09	34.11	45.76	55.95	63.79	68.86	71.53	72.59	72.71	72.52
0.48	24.27	36.34	47.40	56.63	64.10	69.17	72.51	74.52	75.52	76.01
0.50	26.17	37.67	48.37	58.39	66.66	72.57	76.61	78.78	80.11	81.01
0.52	27.29	39.45	51.24	61.67	69.95	76.01	79.92	82.40	84.06	84.98
0.54	28.35	40.61	52.28	62.79	71.67	78.04	82.26	84.80	86.01	86.29
0.56	27.76	41.33	54.45	66.18	75.58	81.96	85.71	87.51	88.07	88.07
0.58	30.05	44.03	56.93	68.09	76.82	82.63	86.14	87.94	88.62	88.76
0.60	33.49	46.37	58.16	68.47	76.90	82.83	86.62	88.75	89.59	89.59
0.62	34.36	47.20	59.32	70.63	79.91	86.31	90.27	91.73	91.78	91.12
0.64	33.82	47.98	61.74	74.00	83.51	89.66	92.88	93.93	93.56	92.50
0.66	34.62	49.40	63.28	75.34	84.64	90.62	93.76	94.77	94.40	93.38
0.68	36.66	51.27	64.86	76.63	85.76	91.69	94.87	95.95	95.57	94.39
0.70	39.32	53.53	66.98	78.76	87.95	93.89	96.85	97.61	96.89	95.46
0.72	39.75	54.26	68.07	80.15	89.49	95.42	98.36	99.17	98.62	97.47
0.74	39.59	54.48	68.55	80.68	89.99	95.99	99.16	100	100	99.49
0.76	41.03	55.81	69.59	81.46	90.74	96.76	100	100	100	99.46
0.78	42.89	57.45	71.12	83.00	92.09	97.61	100	100	99.60	98.22
0.80	43.11	58.44	72.88	84.93	93.10	97.52	99.72	100	99.66	98.51

Table S7. Conversion data from the concentration sweep experiment conducted at 30 °C, extracted from the original 3D surface plot.

Initial monomer concentration / M	*Conversion at different residence times (second) / %*									
	1	2	3	4	5	6	7	8	9	10
0.20	12.40	17.50	22.42	26.99	31.04	34.29	36.73	38.62	40.21	41.73
0.22	15.07	19.72	24.16	28.26	31.89	34.88	37.42	39.63	41.64	43.56
0.24	16.64	21.02	25.19	29.15	32.82	36.04	38.85	41.31	43.56	45.72
0.26	16.93	21.61	26.25	30.84	34.98	38.52	41.62	44.29	46.86	49.38
0.28	18.10	23.27	28.40	33.18	37.27	40.85	43.98	46.88	49.72	52.49
0.30	21.12	25.96	30.51	34.71	38.57	42.19	45.58	48.85	52.15	55.76
0.32	23.78	27.94	32.00	35.99	39.95	43.98	48.19	52.64	57.38	62.45
0.34	25.22	29.22	33.13	37.10	41.27	45.75	50.69	55.99	61.55	67.28
0.36	27.21	30.90	34.49	38.29	42.75	47.85	53.50	59.43	65.25	70.75
0.38	28.66	33.04	37.57	43.12	49.06	54.93	60.84	65.75	70.45	74.89
0.40	29.91	36.22	43.06	49.88	56.16	61.76	66.58	70.81	74.52	77.78
0.42	31.45	38.95	46.46	53.68	60.34	65.82	70.25	73.80	76.53	78.51
0.44	32.14	40.94	49.71	57.86	64.80	70.04	73.75	76.35	78.23	79.79
0.46	33.06	42.44	51.28	59.21	65.86	70.87	74.62	77.32	79.27	80.76
0.48	34.03	43.33	52.17	60.30	67.08	72.23	75.82	78.11	79.57	80.70
0.50	34.82	44.43	53.68	62.01	68.86	73.88	77.32	79.64	81.41	82.70
0.52	36.56	46.33	55.62	63.85	70.65	75.68	79.23	81.62	83.07	84.01
0.54	38.73	48.26	57.31	65.38	71.99	76.75	79.86	81.75	82.85	83.72
0.56	39.60	49.07	58.00	65.96	72.50	77.35	80.79	83.20	84.91	86.31
0.58	41.03	50.41	59.37	67.41	74.05	79.01	82.45	84.74	86.25	87.35
0.60	42.32	51.68	60.59	68.44	74.89	79.60	82.83	84.89	86.02	86.52
0.62	43.81	53.12	61.85	69.74	76.25	80.81	83.68	84.85	84.97	84.67
0.64	44.19	53.71	62.81	71.00	77.35	81.58	83.85	84.73	84.82	84.43
0.66	44.49	54.52	63.94	72.17	78.67	83.09	85.71	86.93	87.10	86.52
0.68	47.23	57.33	67.03	75.62	82.42	86.84	89.08	89.67	89.12	87.95
0.70	47.80	58.45	68.60	77.41	84.16	88.34	90.27	90.51	89.59	88.07
0.72	48.18	59.16	69.43	78.26	85.08	89.34	91.35	91.66	90.62	88.69
0.74	49.60	60.99	71.61	81.14	88.59	93.09	95.07	94.47	92.36	89.55
0.76	50.37	62.61	74.35	84.63	92.21	96.36	97.49	96.41	93.88	90.86
0.78	50.11	63.08	75.21	85.49	92.84	96.76	97.91	97.06	95.19	93.30
0.80	50.61	63.80	75.93	85.83	92.32	95.72	97.39	97.76	97.27	96.35

Table S8. Conversion data from the concentration sweep experiment conducted at 20 °C, extracted from the original 3D surface plot.

Initial monomer concentration / M	*Conversion at different residence times (second) / %*									
	1	2	3	4	5	6	7	8	9	10
0.20	14.42	19.99	25.47	30.78	35.83	40.27	43.92	47.00	49.72	52.28
0.22	15.10	21.13	27.27	33.15	38.49	43.04	46.88	50.13	52.94	55.48
0.24	15.18	22.06	28.90	35.34	41.08	45.89	49.81	52.89	55.30	57.31
0.26	16.58	24.37	31.95	39.22	45.68	50.79	54.73	57.31	59.42	61.23
0.28	18.17	26.82	35.39	43.40	50.12	55.28	59.04	61.81	63.97	65.74
0.30	18.76	28.18	37.28	45.57	52.57	57.99	62.01	64.98	67.24	69.13
0.32	20.24	30.16	39.76	48.58	56.14	62.05	66.47	69.71	72.10	73.97
0.34	22.27	33.04	43.66	53.39	61.60	67.87	72.23	75.07	76.81	77.90
0.36	24.67	36.44	47.81	58.31	67.02	73.41	77.46	79.78	80.92	81.43
0.38	25.66	38.50	50.98	62.37	71.52	77.86	81.81	83.81	84.64	84.93
0.40	27.24	40.96	54.07	65.55	74.58	80.96	84.86	87.02	88.13	88.49
0.42	30.65	44.36	57.31	68.82	78.34	85.05	89.32	91.69	92.60	92.52
0.44	32.70	47.19	61.26	73.86	83.95	90.72	94.59	96.30	96.60	96.26
0.46	33.31	48.65	63.01	75.61	85.64	92.44	96.49	98.24	98.31	97.35
0.48	34.94	50.75	65.74	79.33	89.88	96.87	100	100	100	98.32
0.50	37.50	53.94	69.57	82.99	93.25	99.60	100	100	100	98.85
0.52	39.17	55.68	71.12	84.51	94.87	100	100	100	100	99.32
0.54	39.64	56.48	72.35	86.05	96.29	100	100	100	100	99.48
0.56	40.32	57.10	72.64	85.87	95.74	100	100	100	100	98.40
0.58	41.27	57.71	73.03	86.19	96.10	100	100	100	100	99.18
0.60	42.43	59.01	74.60	87.97	97.77	100	100	100	100	98.71
0.62	45.09	61.17	76.02	88.14	97.07	100	100	100	100	98.84
0.64	46.45	61.53	75.32	87.27	96.49	100	100	100	100	99.88
0.66	46.34	61.78	76.29	88.74	97.90	100	100	100	100	99.42
0.68	49.19	64.20	77.94	89.53	98.07	100	100	100	100	98.14
0.70	51.80	66.08	79.50	91.00	99.51	100	100	100	100	99.22
0.72	51.72	66.10	79.62	90.96	99.27	100	100	100	100	99.46
0.74	51.69	65.92	79.07	90.10	98.24	100	100	100	100	99.46
0.76	52.58	66.43	79.22	90.12	98.28	100	100	100	100	99.34
0.78	53.39	67.21	80.08	91.02	98.96	100	100	100	100	98.81
0.80	54.48	68.63	81.69	92.31	99.10	100	100	100	100	99.14

Table S9. Conversion data from the concentration sweep experiment conducted at 10 °C, extracted from the original 3D surface plot.

Initial monomer concentration / M	*Conversion at different residence times (second) / %*									
	1	2	3	4	5	6	7	8	9	10
0.20	24.90	30.68	36.34	41.78	46.88	51.27	54.77	57.63	60.06	62.30
0.22	27.66	33.78	39.91	45.65	50.71	54.85	58.21	61.02	63.53	65.94
0.24	30.29	36.62	42.74	48.21	53.06	57.09	60.60	63.69	66.37	68.76
0.26	33.78	39.95	45.76	51.25	56.23	60.44	63.97	66.68	68.90	70.87
0.28	36.63	42.94	49.11	54.98	60.08	64.18	67.33	69.78	71.74	73.44
0.30	38.86	45.77	52.46	58.63	63.91	68.14	71.40	73.91	75.83	77.33
0.32	42.24	49.43	56.54	63.15	68.88	73.32	76.53	78.79	80.39	81.60
0.34	43.59	51.71	59.73	67.02	73.08	77.58	80.66	82.65	83.86	84.65
0.36	45.49	54.24	62.55	70.12	76.50	81.26	84.44	86.45	87.53	87.98
0.38	46.70	56.25	65.48	74.25	81.57	86.77	90.17	91.63	92.07	92.07
0.40	47.22	58.20	69.00	78.74	86.39	91.56	94.53	95.92	96.40	96.40
0.42	48.79	60.46	71.49	81.19	88.87	94.15	97.35	99.00	99.59	99.57
0.44	50.87	62.58	73.69	83.52	91.40	96.76	99.80	100	100	99.97
0.46	52.22	64.48	76.25	86.50	94.35	99.27	100	100	100	98.47
0.48	54.44	66.85	78.39	88.17	95.59	100	100	100	100	98.06
0.50	56.38	68.58	79.88	89.77	97.29	100	100	100	100	98.12
0.52	57.28	69.76	81.54	91.69	99.15	100	100	100	100	98.99
0.54	57.90	70.52	82.25	92.22	99.54	100	100	100	100	99.64
0.56	58.88	71.27	82.67	92.25	99.20	100	100	100	100	98.22
0.58	60.12	71.82	82.49	91.52	98.22	100	100	100	100	99.10
0.60	60.41	71.87	82.58	91.83	98.70	100	100	100	100	99.38
0.62	61.09	72.70	83.56	92.76	99.50	100	100	100	100	98.70
0.64	62.17	73.51	83.99	92.85	99.22	100	100	100	100	99.09
0.66	62.96	73.90	83.95	92.43	98.68	100	100	100	100	99.75
0.68	63.60	74.21	84.14	92.66	99.04	100	100	100	100	98.81
0.70	63.08	74.01	84.30	93.07	99.52	100	100	100	100	99.23
0.72	63.47	74.53	84.81	93.40	99.64	100	100	100	100	98.96
0.74	64.85	75.38	85.00	92.97	98.91	100	100	100	100	99.38
0.76	64.36	74.57	84.04	92.33	98.70	100	100	100	100	99.80
0.78	62.76	73.74	84.10	92.99	99.39	100	100	100	100	99.17
0.80	64.03	75.33	85.66	93.99	99.29	100	100	100	100	99.22

Table S10. Conversion data from the concentration sweep experiment conducted at 0 °C, extracted from the original 3D surface plot.

Initial monomer concentration / M	*Conversion at different residence times (second) / %*									
	1	2	3	4	5	6	7	8	9	10
0.20	28.52	37.40	45.82	53.29	59.37	63.44	65.57	66.31	66.16	65.67
0.22	38.68	45.90	52.51	58.19	62.60	65.39	66.87	67.49	67.60	67.52
0.24	44.90	50.80	56.14	60.70	64.39	66.88	68.48	69.49	70.12	70.62
0.26	47.62	53.02	58.08	62.65	66.38	69.13	71.11	72.57	73.70	74.78
0.28	50.52	55.85	60.92	65.33	69.03	72.06	74.42	76.38	78.24	80.06
0.30	53.69	58.58	63.28	67.67	71.71	75.20	78.19	80.83	83.21	85.37
0.32	53.93	59.32	64.78	70.06	74.89	78.98	82.33	85.14	87.59	89.85
0.34	53.60	60.13	66.77	73.08	78.72	83.42	87.33	90.64	93.52	96.18
0.36	53.96	61.57	69.11	76.18	82.41	87.57	91.78	95.07	97.55	99.46
0.38	55.31	63.86	72.20	80.07	86.86	92.08	95.89	98.18	99.48	100
0.40	57.58	67.14	76.51	84.94	92.05	96.93	99.88	100	100	99.83
0.42	59.34	69.82	80.00	89.11	96.28	100	100	100	100	99.31
0.44	59.53	71.02	81.90	91.30	98.35	100	100	100	100	99.16
0.46	60.13	71.77	82.66	92.01	98.99	100	100	100	100	99.88
0.48	59.64	71.53	82.77	92.29	99.25	100	100	100	100	99.44
0.50	61.18	72.96	83.80	92.76	99.35	100	100	100	100	99.07
0.52	63.01	74.04	84.15	92.77	99.13	100	100	100	100	98.90
0.54	62.70	73.68	83.90	92.62	99.06	100	100	100	100	98.41
0.56	63.05	74.11	84.40	93.10	99.45	100	100	100	100	98.29
0.58	64.00	74.88	84.83	93.19	99.28	100	100	100	100	98.15
0.60	65.30	75.74	85.32	93.58	99.70	100	100	100	100	99.23
0.62	64.74	75.25	85.15	93.68	99.91	100	100	100	100	99.87
0.64	64.28	75.08	85.15	93.60	99.77	100	100	100	100	98.97
0.66	65.42	75.94	85.66	93.87	99.90	100	100	100	100	98.49
0.68	65.85	76.25	85.86	93.97	99.84	100	100	100	100	99.41
0.70	66.84	76.90	86.04	93.70	99.30	100	100	100	100	98.36
0.72	68.09	77.61	86.31	93.89	99.49	100	100	100	100	97.83
0.74	66.97	76.70	85.94	93.95	99.71	100	100	100	100	98.75
0.76	66.64	76.80	86.28	94.06	99.76	100	100	100	100	99.10
0.78	67.49	77.26	86.36	94.14	99.90	100	100	100	100	98.59
0.80	64.03	74.98	85.45	94.13	99.69	100	100	100	100	99.25

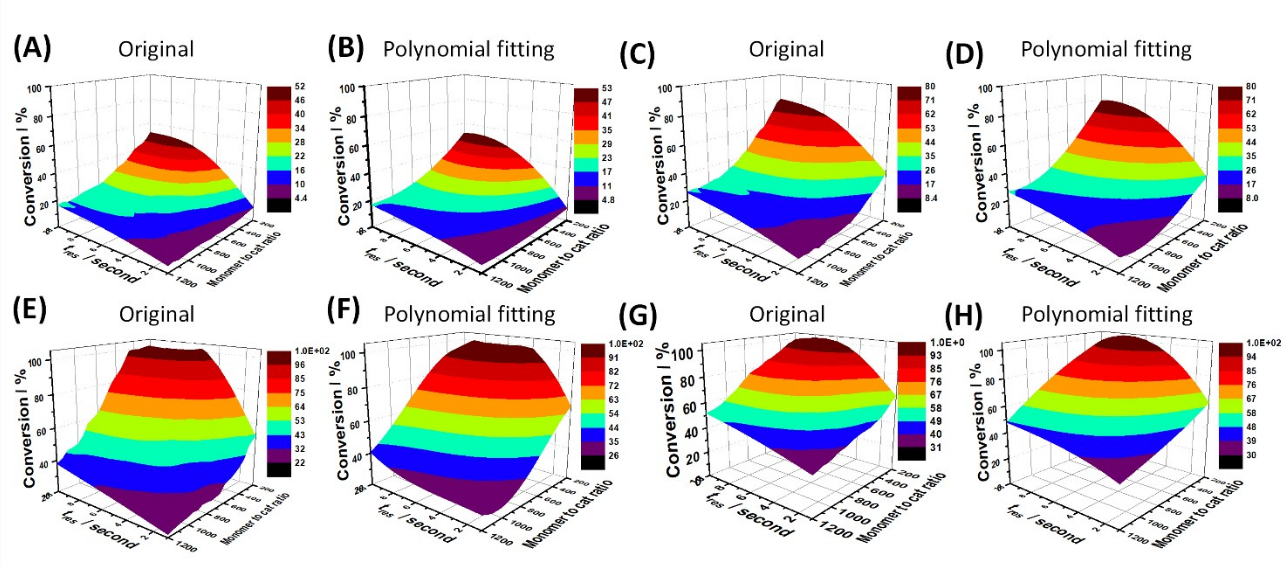

Figure S17. 3-dimensional surface plots of experimental data from monomer-to-catalyst ratio sweep experiments conducted at 20 °C with initial monomer concentration of (A) 0.2 M, (C) 0.35 M, (E) 0.5 M, and (G) 0.7 M; and corresponding three-dimensional surface plots from polynomial fitting with initial monomer concentration of (B) 0.2 M, (D) 0.35 M, (F) 0.5 M, and (H) 0.7 M.

Table S11. 3-dimensional fitting results of monomer-to-catalyst ratio sweep data.

C_m / (mol·L^{-1})	*A*	*B*	*C*	*D*	*E*	*F*	*G*	*H*	*I*	*J*	$R^{2\,a}$	$R^{2\,b}$
0.2	0	0.007	0.001	0	0	-0.99	-0.015	0.004	16.212	-3.512	0.996	0.996
0.35	0	0.008	0.001	0	0	-0.997	-0.013	-0.016	16.268	25.445	0.996	0.995
0.5	0	0.028	0.001	0	0	-2.073	-0.009	0.012	24.291	30.414	0.989	0.989
0.7	0	0.013	0.001	0	0	-1.418	-0.008	-0.040	18.128	51.675	0.997	0.996

a: is the R^2 value of fitting between the 3-dimensional polynomial fitting plots and the original 3-dimensional surface plot; b: is the R^2 value between the experimental data (shown in Figures S11 and S12) and the polynomial fitting.

Table S12. Conversion data from the monomer-to-catalyst ratio sweep experiment, conducted at 20 °C with an initial monomer concentration of 0.2 M, extracted from the original 3D surface plot.

Monomer-to-catalyst ratio	*Conversion at different residence times (second) / %*									
	1	2	3	4	5	6	7	8	9	10
200	10.38	19.94	28.95	36.89	43.21	47.55	50.15	51.47	51.93	51.95
250	9.60	18.56	26.99	34.39	40.22	44.12	46.36	47.36	47.53	47.32
300	8.90	17.98	26.51	33.92	39.53	43.12	44.91	45.36	44.92	44.23
350	8.03	16.55	24.59	31.61	36.96	40.50	42.43	43.16	43.17	42.90
400	7.37	15.85	23.88	30.75	35.85	39.22	40.92	41.40	41.44	40.99
450	6.99	14.49	21.59	27.50	32.21	35.22	36.77	37.29	37.64	37.06
500	6.92	14.07	20.86	26.21	30.60	33.19	34.25	34.43	34.47	33.50
550	6.69	13.18	19.34	23.99	27.96	30.34	31.29	31.61	31.80	31.31
600	6.12	11.83	17.22	21.43	25.13	27.51	28.81	29.78	30.19	30.06
650	6.03	11.10	15.79	19.54	22.80	24.86	25.95	26.90	27.15	27.04
700	6.18	10.86	15.07	18.70	21.73	23.59	24.62	25.54	25.48	25.18
750	5.49	10.24	14.39	18.19	21.17	22.74	23.54	24.02	23.27	22.38
800	5.74	10.22	13.80	17.02	19.58	20.88	21.51	21.86	21.64	21.18
850	6.01	9.63	12.63	15.45	17.71	19.11	20.13	20.62	20.68	20.60
900	6.05	9.17	11.89	14.44	16.51	17.86	18.94	19.51	19.76	19.90
950	5.57	8.52	11.21	13.67	15.69	17.09	18.23	18.86	19.24	19.49
1000	5.46	8.55	11.53	14.16	16.19	17.56	18.38	18.57	18.44	18.19
1050	4.73	7.80	10.74	13.29	15.28	16.56	17.24	17.43	17.32	17.05
1100	4.51	7.15	9.64	11.84	13.61	14.82	15.54	15.91	16.06	16.08
1150	5.42	7.23	8.98	10.63	12.12	13.41	14.54	15.56	16.49	17.37
1200	4.68	6.56	8.38	10.07	11.58	12.87	13.96	14.90	15.74	16.53

Table S13. Conversion data from the monomer-to-catalyst ratio sweep experiment, conducted at 20 °C with an initial monomer concentration of 0.35 M, extracted from the original 3D surface plot.

Monomer-to-catalyst ratio	*Conversion at different residence times (second) / %*									
	1	2	3	4	5	6	7	8	9	10
200	36.95	45.89	54.39	61.98	68.21	72.78	75.89	77.92	79.23	80.18
250	33.89	42.57	50.83	58.25	64.40	68.99	72.24	74.49	76.07	77.35
300	29.87	38.50	46.71	54.05	60.02	64.37	67.33	69.25	70.49	71.45
350	27.74	36.77	45.30	52.81	58.60	62.52	64.78	65.81	66.13	66.13
400	25.12	34.05	42.48	49.69	55.10	58.63	60.37	60.81	60.81	60.29
450	22.85	31.30	39.33	46.04	51.39	54.92	56.83	57.62	58.23	57.92
500	20.28	28.60	36.49	42.91	48.27	51.66	53.40	54.22	54.86	54.29
550	17.43	25.81	33.74	39.96	45.28	48.37	49.70	50.31	50.44	49.32
600	15.67	23.86	31.50	37.37	42.39	45.18	46.09	46.60	46.29	45.07
650	13.41	21.20	28.36	33.98	38.82	41.56	42.70	43.60	43.39	42.57
700	12.99	19.87	25.96	30.87	35.00	37.42	38.48	39.38	39.07	38.42
750	12.03	18.05	23.25	28.04	31.92	34.19	35.48	36.43	36.25	35.81
800	11.35	16.60	20.89	24.76	28.04	30.11	31.46	32.63	33.34	33.77
850	11.31	15.25	18.53	21.65	24.36	26.43	28.13	29.43	30.42	31.29
900	11.19	14.29	17.14	19.80	22.16	24.05	25.77	27.10	28.21	29.24
950	9.60	13.27	16.91	20.28	23.03	25.13	26.88	27.85	28.49	29.03
1000	8.51	12.57	16.46	19.97	22.82	24.83	26.32	27.16	27.65	27.97
1050	10.18	13.48	16.53	19.31	21.66	23.45	24.78	25.75	26.49	27.11
1100	8.94	12.43	15.81	18.83	21.32	23.19	24.44	25.25	25.79	26.19
1150	9.60	12.43	15.16	17.71	19.98	21.89	23.51	24.91	26.17	27.34
1200	9.34	11.92	14.42	16.76	18.86	20.66	22.20	23.55	24.78	25.93

Table S14. Conversion data from the monomer-to-catalyst ratio sweep experiment, conducted at 20 °C with an initial monomer concentration of 0.5 M, extracted from the original 3D surface plot

Monomer-to-catalyst ratio	*Conversion at different residence times (second) / %*									
	1	2	3	4	5	6	7	8	9	10
200	52.09	66.56	80.02	91.49	99.95	100	100	100	100	100
250	50.37	65.33	79.25	91.08	99.73	100	100	100	100	100
300	47.57	63.19	77.77	90.21	99.23	100	100	100	100	100
350	41.00	56.73	71.52	84.27	93.61	99.39	100	100	100	100
400	38.06	53.78	68.65	81.32	90.80	96.94	99.92	100	100	100
450	35.75	50.36	64.36	75.99	85.26	91.66	95.36	97.14	98.59	98.99
500	33.92	46.49	58.61	68.49	76.86	82.97	86.91	89.55	91.96	93.21
550	32.79	43.80	54.30	63.12	71.06	76.71	80.59	83.68	86.18	87.40
600	31.39	42.15	52.34	60.73	68.32	73.63	77.01	79.98	82.32	83.81
650	27.46	37.45	46.90	54.63	61.69	67.04	70.92	74.51	77.34	79.62
700	26.83	35.51	43.55	50.78	57.20	62.09	65.92	69.46	71.58	73.40
750	26.26	34.46	41.83	48.69	54.56	58.70	61.78	64.58	66.06	67.26
800	26.81	34.53	41.20	47.39	52.57	56.11	58.82	60.81	61.66	62.34
850	27.06	33.89	39.88	45.61	50.37	53.56	56.19	57.92	58.87	59.61
900	25.37	31.51	36.85	42.01	46.39	49.51	52.20	54.25	55.86	57.17
950	25.81	30.22	33.95	37.48	40.66	43.23	45.45	47.26	48.86	50.35
1000	24.75	28.36	31.98	35.34	38.23	40.59	42.50	43.92	45.09	46.18
1050	24.21	27.00	29.63	32.19	34.65	36.82	38.94	40.99	42.97	44.91
1100	23.53	25.77	28.02	30.27	32.53	34.78	37.07	39.37	41.67	43.97
1150	22.79	24.38	26.00	27.70	29.49	31.41	33.45	35.56	37.74	39.94
1200	21.70	23.62	25.53	27.41	29.24	31.02	32.75	34.44	36.11	37.77

Table S15. Conversion data from the monomer-to-catalyst ratio sweep experiment, conducted at 20 °C with an initial monomer concentration of 0.7 M, extracted from the original 3D surface plot

Monomer-to-catalyst ratio	*Conversion at different residence times (second) / %*									
	1	2	3	4	5	6	7	8	9	10
200	62.23	72.69	82.44	90.81	97.11	100	100	100	100	100
250	60.83	71.63	81.70	90.30	96.66	100	100	100	100	100
300	57.02	67.78	77.86	86.55	93.00	96.92	98.62	98.66	97.59	96.15
350	53.66	64.33	74.34	82.97	89.34	93.28	95.03	95.15	94.32	93.11
400	50.61	61.44	71.66	80.32	86.68	90.62	92.30	92.34	91.80	90.50
450	48.80	59.34	69.32	77.52	83.98	87.88	89.58	89.79	89.77	88.38
500	47.38	57.81	67.75	75.49	81.77	85.52	87.00	87.17	87.22	86.02
550	45.67	55.45	64.72	72.03	78.32	82.18	84.07	85.09	85.62	84.85
600	45.03	54.62	63.62	70.68	76.79	80.40	82.00	83.13	83.19	82.21
650	44.57	53.63	61.94	68.50	74.16	77.49	78.93	80.17	80.17	79.50
700	43.75	52.15	59.65	66.02	71.42	74.77	76.61	78.25	78.45	78.20
750	42.60	50.59	57.56	63.85	69.05	72.29	74.31	75.97	76.20	76.10
800	41.11	48.74	55.28	61.38	66.31	69.33	71.44	72.78	72.85	72.68
850	39.23	46.35	52.41	58.09	62.66	65.45	67.59	68.69	68.89	68.82
900	38.50	44.99	50.59	55.83	59.96	62.51	64.48	65.15	65.10	64.83
950	37.34	43.18	48.45	53.28	57.10	59.48	61.28	61.95	62.07	61.93
1000	35.23	40.55	45.51	49.98	53.57	56.00	57.71	58.55	58.94	59.09
1050	34.01	38.48	42.70	46.50	49.65	51.96	53.57	54.63	55.35	55.89
1100	33.75	37.43	40.95	44.17	46.93	49.09	50.75	52.03	53.08	54.00
1150	32.05	35.17	38.20	41.05	43.63	45.87	47.82	49.56	51.16	52.68
1200	30.81	33.65	36.44	39.12	41.64	43.96	46.10	48.11	50.03	51.91

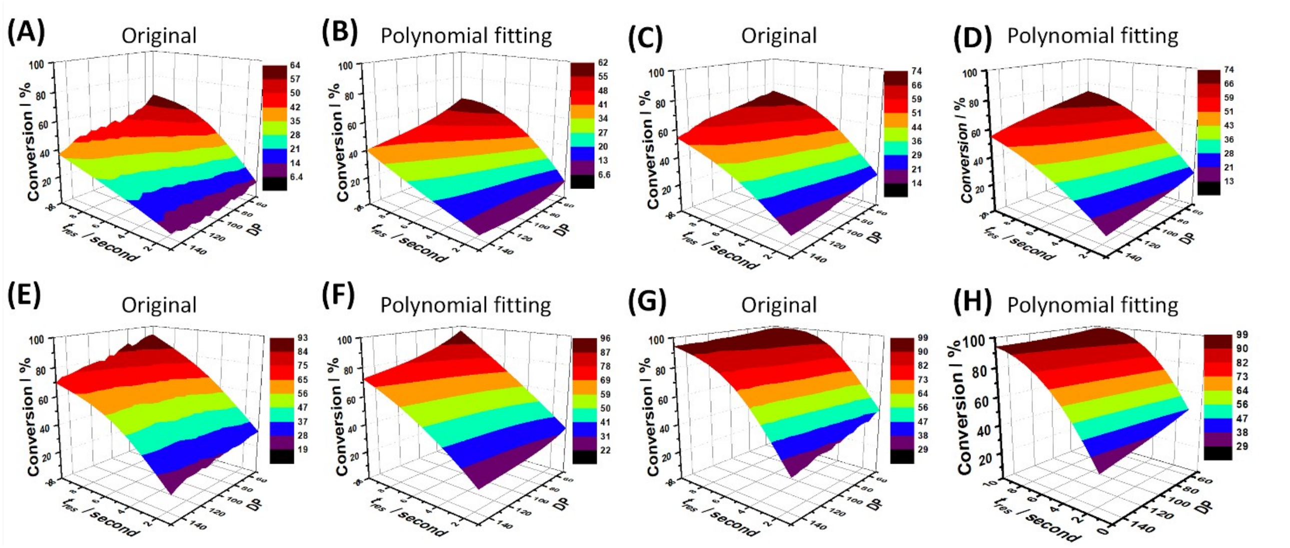

Figure S18. 3-dimensional surface plots of experimental data from degree of polymerization sweep experiments conducted at 20 °C with initial monomer concentration of (A) 0.2 M, (C) 0.35 M, (E) 0.5 M, and (G) 0.7 M; and corresponding three-dimensional surface plots from polynomial fitting with initial monomer concentration of (B) 0.2 M, (D) 0.35 M, (F) 0.5 M, and (H) 0.7 M.

Table S16. 3-dimensional fitting results of degree of polymerization sweep data.

C_m / (mol·L^{-1})	*A*	*B*	*C*	*D*	*E*	*F*	*G*	*H*	*I*	*J*	$R^{2\,a}$	$R^{2\,b}$
0.2	0	0.007	0.008	0	0.006	-1.219	-0.095	-0.709	18.679	21.211	0.995	0.994
0.35	0	0.007	0.002	0	0.003	-0.772	-0.016	-0.385	12.789	26.898	0.998	0.999
0.5	0	0.003	-0.002	0	0.005	-0.064	-0.009	-0.645	8.501	47.841	0.996	0.996
0.7	0	0.014	0.002	0	0.001	-1.232	-0.009	-0.260	17.089	42.358	0.999	0.999

a: is the R^2 value of fitting between the 3-dimensional polynomial fitting plots and the original 3-dimensional surface plot; b: is the R^2 value between the experimental data (shown in Figures S13 and S14) and the polynomial fitting.

Table S17. Conversion data from the Degree of polymerization sweep experiment, conducted at 20 °C with an initial monomer concentration of 0.2 M, extracted from the original 3D surface plot.

Degree of polymerization	*Conversion at different residence times (second) / %*									
	1	2	3	4	5	6	7	8	9	10
50	11.54	21.98	31.93	40.93	48.51	54.20	58.15	60.79	62.55	63.88
55	10.97	21.35	31.20	39.99	47.15	52.35	55.87	58.12	59.49	60.36
60	11.29	21.52	31.19	39.72	46.49	51.16	54.08	55.68	56.41	56.70
65	8.60	18.71	28.21	36.56	43.20	47.85	50.88	52.74	53.84	54.62
70	7.56	17.07	26.00	33.90	40.33	45.08	48.48	50.87	52.59	53.99
75	8.57	17.21	25.39	32.71	38.74	43.23	46.40	48.58	50.09	51.26
80	7.63	16.44	24.76	32.16	38.20	42.68	45.93	48.29	50.08	51.63
85	9.91	17.39	24.49	30.89	36.28	40.47	43.63	45.99	47.80	49.28
90	7.79	15.68	23.18	29.90	35.41	39.50	42.44	44.55	46.15	47.56
95	7.72	14.76	21.46	27.58	32.87	37.27	40.99	44.25	47.25	50.19
100	6.55	13.18	19.52	25.39	30.64	35.20	39.18	42.72	45.94	48.97
105	8.59	14.41	20.06	25.37	30.16	34.29	37.86	41.01	43.91	46.70
110	6.60	12.65	18.50	23.99	28.97	33.41	37.46	41.23	44.83	48.37
115	8.55	13.69	18.71	23.50	27.99	32.10	35.89	39.44	42.86	46.22
120	6.64	11.75	16.77	21.64	26.28	30.70	34.96	39.09	43.15	47.17
125	7.22	12.15	16.96	21.58	25.96	30.04	33.86	37.49	40.98	44.39
130	8.73	13.00	17.22	21.39	25.46	29.46	33.43	37.40	41.40	45.45
135	7.50	11.68	15.83	19.95	24.05	28.12	32.12	36.05	39.88	43.61
140	8.17	12.63	17.01	21.22	25.17	28.79	32.08	35.11	37.94	40.63
145	10.20	14.30	18.28	22.03	25.44	28.47	31.18	33.65	35.99	38.27
150	9.94	13.42	16.80	20.05	23.12	26.01	28.77	31.45	34.06	36.64

Table S18. Conversion data from the Degree of polymerization sweep experiment, conducted at 20 °C with an initial monomer concentration of 0.35 M, extracted from the original 3D surface plot.

Degree of polymerization	*Conversion at different residence times (second) / %*									
	1	2	3	4	5	6	7	8	9	10
50	23.42	32.96	42.10	50.43	57.56	63.07	67.08	69.97	72.10	73.85
55	23.24	32.84	42.01	50.26	57.10	62.25	65.98	68.68	70.69	72.40
60	22.98	32.09	40.73	48.49	54.99	60.03	63.92	66.94	69.42	71.65
65	22.72	31.35	39.60	47.13	53.59	58.79	62.92	66.26	69.04	71.52
70	22.32	31.24	39.78	47.53	54.07	59.17	63.06	66.06	68.47	70.60
75	21.87	30.51	38.74	46.21	52.55	57.56	61.46	64.54	67.05	69.28
80	21.10	29.75	38.02	45.51	51.85	56.83	60.71	63.79	66.37	68.74
85	20.44	28.50	36.19	43.22	49.33	54.36	58.51	62.00	65.03	67.82
90	19.88	27.93	35.68	42.81	48.98	54.01	58.10	61.49	64.42	67.14
95	19.37	27.26	34.83	41.79	47.86	52.86	56.99	60.47	63.51	66.32
100	18.69	26.57	34.14	41.11	47.18	52.17	56.27	59.71	62.72	65.50
105	18.19	25.95	33.43	40.33	46.36	51.38	55.54	59.07	62.17	65.06
110	17.09	25.00	32.61	39.63	45.74	50.77	54.92	58.40	61.45	64.28
115	16.61	24.38	31.84	38.72	44.73	49.74	53.93	57.51	60.69	63.69
120	16.21	23.73	30.97	37.67	43.60	48.61	52.87	56.56	59.89	63.04
125	15.69	23.10	30.26	36.91	42.81	47.83	52.11	55.84	59.18	62.33
130	15.08	22.51	29.68	36.32	42.16	47.04	51.11	54.55	57.57	60.35
135	14.60	22.13	29.38	36.05	41.85	46.61	50.50	53.72	56.50	59.03
140	14.27	21.73	28.87	35.38	40.96	45.44	49.01	51.91	54.38	56.63
145	14.12	21.05	27.68	33.75	39.02	43.39	47.05	50.18	52.97	55.62
150	13.89	20.53	26.90	32.76	37.86	42.10	45.69	48.78	51.54	54.13

Table S19. Conversion data from the Degree of polymerization sweep experiment, conducted at 20 °C with an initial monomer concentration of 0.5 M, extracted from the original 3D surface plot.

Degree of polymerization	*Conversion at different residence times (second) / %*									
	1	2	3	4	5	6	7	8	9	10
50	31.35	40.62	49.63	58.11	65.82	72.52	78.34	83.53	88.34	93.03
55	30.97	39.51	47.82	55.75	63.13	69.87	76.08	81.89	87.43	92.84
60	30.96	39.36	47.58	55.43	62.70	69.30	75.34	81.00	86.41	91.75
65	29.99	37.82	45.46	52.82	59.78	66.31	72.52	78.49	84.30	90.06
70	29.06	36.42	43.65	50.67	57.40	63.81	69.98	75.97	81.85	87.71
75	28.32	35.12	41.84	48.47	55.03	61.47	67.82	74.09	80.30	86.46
80	28.38	35.45	42.49	49.42	56.19	62.78	69.29	75.76	82.26	88.85
85	28.75	34.93	41.09	47.30	53.64	60.15	66.76	73.42	80.07	86.65
90	27.83	34.58	41.35	48.06	54.63	60.98	67.12	73.07	78.89	84.59
95	27.82	34.59	41.28	47.79	54.02	59.91	65.51	70.90	76.14	81.30
100	26.90	33.75	40.51	47.11	53.48	59.59	65.48	71.18	76.74	82.17
105	27.29	34.34	41.30	48.00	54.27	59.95	65.09	69.81	74.24	78.49
110	25.29	32.64	39.84	46.70	53.06	58.84	64.21	69.29	74.24	79.19
115	24.25	31.15	37.86	44.33	50.51	56.41	62.10	67.65	73.11	78.53
120	25.49	31.63	37.73	43.76	49.71	55.54	61.23	66.79	72.25	77.60
125	24.35	31.19	37.98	44.58	50.85	56.68	62.11	67.25	72.18	76.99
130	24.15	31.24	38.18	44.82	51.00	56.64	61.82	66.63	71.19	75.59
135	24.15	31.31	38.32	45.01	51.17	56.68	61.62	66.11	70.27	74.22
140	22.73	30.55	38.18	45.34	51.77	57.29	62.01	66.13	69.83	73.29
145	21.14	29.58	37.75	45.34	52.00	57.55	62.16	66.08	69.54	72.75
150	18.76	27.99	36.86	44.90	51.67	56.96	61.11	64.39	67.09	69.51

Table S20. Conversion data from the Degree of polymerization sweep experiment, conducted at 20 °C with an initial monomer concentration of 0.7 M, extracted from the original 3D surface plot.

Degree of polymerization	*Conversion at different residence times (second) / %*									
	1	2	3	4	5	6	7	8	9	10
50	47.19	59.42	70.98	81.18	89.34	94.81	97.80	98.95	98.91	98.31
55	46.85	58.81	70.12	80.02	87.80	93.01	96.09	97.59	98.05	98.04
60	44.86	56.76	67.97	77.82	85.63	91.03	94.45	96.39	97.35	97.85
65	42.57	54.74	66.21	76.30	84.34	89.96	93.57	95.67	96.77	97.37
70	43.13	55.10	66.37	76.30	84.21	89.73	93.28	95.35	96.44	97.06
75	42.75	54.59	65.76	75.61	83.49	89.03	92.63	94.77	95.94	96.60
80	41.83	53.78	65.11	75.12	83.13	88.75	92.39	94.54	95.70	96.39
85	39.76	52.07	63.70	73.94	82.14	87.92	91.71	94.01	95.32	96.14
90	39.82	51.97	63.45	73.59	81.75	87.54	91.35	93.69	95.02	95.85
95	38.68	51.07	62.82	73.22	81.56	87.44	91.25	93.53	94.78	95.53
100	37.00	49.76	61.78	72.37	80.81	86.74	90.63	92.99	94.37	95.27
105	37.43	49.54	61.00	71.19	79.47	85.50	89.65	92.41	94.23	95.58
110	35.07	47.70	59.68	70.33	78.97	85.21	89.46	92.23	94.01	95.31
115	34.91	47.42	59.23	69.71	78.22	84.41	88.71	91.60	93.58	95.14
120	35.21	47.13	58.40	68.44	76.68	82.82	87.25	90.41	92.75	94.69
125	35.08	46.46	57.27	67.00	75.12	81.33	85.98	89.44	92.10	94.37
130	33.75	45.34	56.43	66.43	74.79	81.19	85.94	89.47	92.19	94.50
135	31.83	43.90	55.40	65.77	74.43	81.06	85.97	89.58	92.29	94.54
140	31.52	44.02	55.93	66.60	75.38	81.92	86.62	89.95	92.40	94.46
145	30.18	42.38	53.95	64.36	73.03	79.70	84.75	88.58	91.62	94.28
150	29.57	42.47	54.77	65.74	74.68	81.31	86.15	89.65	92.25	94.41

First-order linear plots for concentration-sweep experiments

To ensure the reliability and accuracy of the first-order kinetic analysis while incorporating all experimental data points into the fitting, only systems that did not reach full conversion (100%) within 10 seconds were examined.

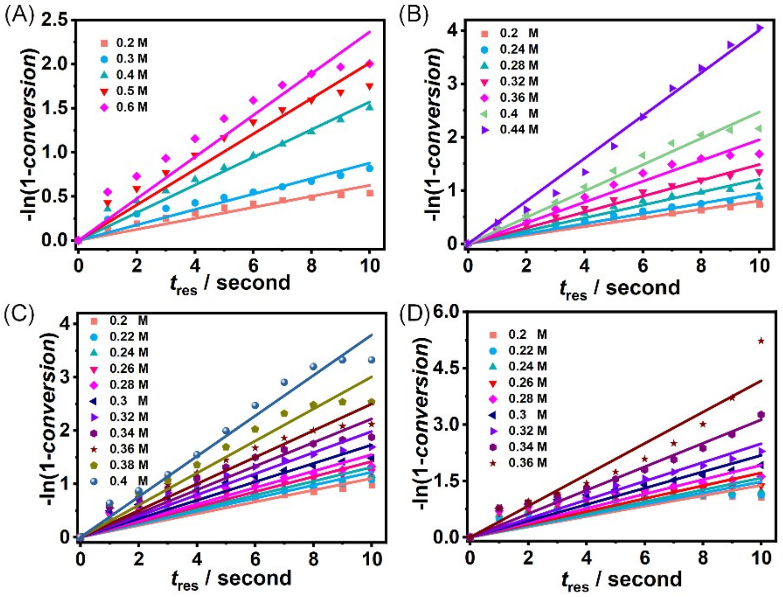

Figure S19. Plots of ln(M_0/M_t) versus residence time of the ROP of L-lactide from concentration-sweep experiments conducted at (A) 30 ˚C, (B) 20 ˚C, (C) 10 ˚C and (D) 0 ˚C.

Table S21. First-order fitting summary of concentration sweep data.

35 °C			30 °C			20 °C			10 °C			0 °C		
C_m / mol/L	K_{obs}	R^2	C_m / mol/L	K_{obs}	R^2	C_m / mol/L	K_{obs}	R^2	C_m / mol/L	K_{obs}	R^2	C_m / mol/L	K_{obs}	R^2
0.2	0.0365	0.94	0.2	0.0625	0.98	0.2	0.0805	0.99	0.2	0.1103	0.99	0.2	0.1726	0.98
0.3	0.077	0.93	0.3	0.0877	0.98	0.24	0.0945	0.99	0.22	0.1219	0.97	0.22	0.189	0.95
0.4	0.129	0.97	0.4	0.1571	0.99	0.28	0.1209	0.99	0.24	0.1312	0.97	0.24	0.2007	0.93
0.5	0.1914	0.99	0.5	0.2012	0.98	0.32	0.1486	0.99	0.26	0.1419	0.97	0.26	0.1973	0.93
0.6	0.2632	0.99	0.6	0.2365	0.98	0.36	0.1951	0.99	0.28	0.1548	0.97	0.28	0.1918	0.94
			0.7	0.3589	0.99	0.4	0.2471	0.99	0.3	0.1729	0.97	0.3	0.2179	0.96
			0.8	0.5149	0.99	0.44	0.4004	0.99	0.32	0.1982	0.97	0.32	0.2489	0.97
									0.34	0.2218	0.97	0.34	0.3126	0.99
									0.36	0.2498	0.98	0.36	0.3792	0.99
									0.38	0.3313	0.99			
									0.4	0.3956	0.99			

First-order linear plots for degree of polymerization sweep experiments

To ensure the reliability and accuracy of the first-order kinetic analysis while incorporating all experimental data points into the fitting, only systems that did not reach full conversion (100%) within 10 seconds were examined.

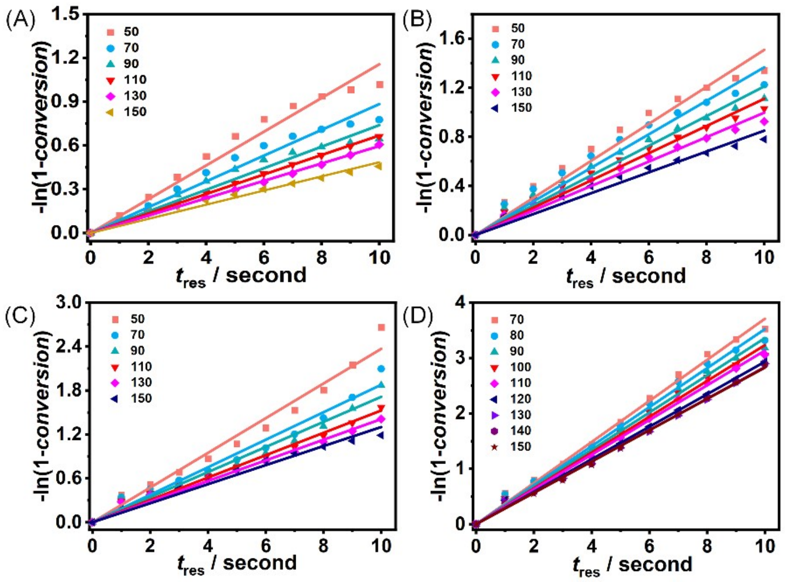

Figure S20. Plots of ln(M_0/M_t) versus residence time of the ROP of L-lactide from degree of polymerization sweep experiments conducted at 20 ˚C with different initial monomer concentrations (A) 0.2 mol/L, (B) 0.35 mol/L, (C) 0.5 mol/L and (D) 0.7 mol/L.

Table S22. First-order fitting summary of degree of polymerization sweep data.

0.2 mol/L			0.35 mol/L			0.5 mol/L			0.7 mol/L		
DP	K'_{obs}	R^2	*DP*	K'_{obs}	R^2	*DP*	K'_{obs}	R^2	*DP*	K'_{obs}	R^2
50	0.1156	0.99	50	0.1509	0.99	50	0.2369	0.99	70	0.3710	0.99
70	0.0833	0.99	70	0.1367	0.99	70	0.1878	0.99	80	0.3523	0.99
90	0.0739	0.99	90	0.1211	0.99	90	0.1714	0.99	90	0.3360	0.99
110	0.0668	0.99	110	0.1110	0.99	110	0.1527	0.99	100	0.3234	0.99
130	0.0596	0.99	130	0.0997	0.99	130	0.1409	0.99	110	0.3135	0.99
150	0.0485	0.99	150	0.0850	0.99	150	0.1301	0.99	120	0.2934	0.99
									130	0.2833	0.99
									140	0.2863	0.99
									150	0.2828	0.99

First-order linear plots for monomer-to-catalyst ratio sweep experiments

To ensure the reliability and accuracy of the first-order kinetic analysis while incorporating all experimental data points into the fitting, only systems that did not reach full conversion (100%) within 10 seconds were examined.

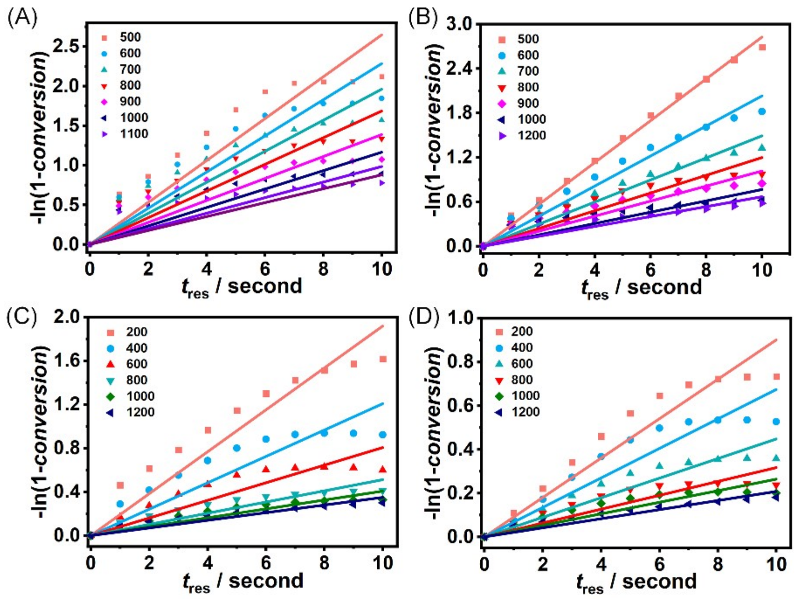

Figure S21. Plots of ln(M_0/M_t) versus residence time of the ROP of L-lactide from monomer-to-catalyst ratio sweep experiments conducted at 20 ˚C with different initial monomer concentrations (A) 0.7 mol/L, (B) 0.5 mol/L, (C) 0.35 mol/L and (D) 0.2 mol/L.

Table S23. First-order fitting summary of monomer-to-catalyst ratio sweep data.

0.2 mol/L			0.35 mol/L			0.5 mol/L			0.7 mol/L		
Monomer to catalyst ratio	K''_{obs}	R^2	*Monomer to catalyst ratio*	K''_{obs}	R^2	*Monomer to catalyst ratio*	K''_{obs}	R^2	*Monomer to catalyst ratio*	K''_{obs}	R^2
200	0.0901	0.98	200	0.1918	0.97	500	0.2825	0.99	500	0.2648	0.96
400	0.0674	0.96	400	0.1207	0.95	600	0.203	0.99	600	0.2284	0.96
600	0.0448	0.97	600	0.0806	0.94	700	0.1491	0.99	700	0.1961	0.95
800	0.0317	0.94	800	0.0512	0.96	800	0.1197	0.96	800	0.1685	0.94
1000	0.0264	0.95	1000	0.0407	0.96	900	0.1015	0.96	900	0.1388	0.93
1200	0.0208	0.98	1200	0.035	0.97	1000	0.0766	0.93	1000	0.1165	0.92
						1100	0.0668	0.94	1100	0.0984	0.91
									1200	0.0879	0.93

Deprotonation of 4-methylbenzyl alcohol by TBD

After being captured by TBD through hydrogen bonding, 4-methylbenzyl alcohol undergoes partial deprotonation. The resulting alkoxide then reacts with the activated L-lactide to initiate ring opening and facilitate chain extension. To better understand the role of the initiator during ring-opening polymerization, the extent of 4-methylbenzyl alcohol deprotonation is estimated in the following section. The conjugated acid of TBD is $TBDH^+$, and its pKa value in acetonitrile is 26.03.[4] The pKa value of alcohol in acetonitrile is 27.7.[5] The reaction equations for the dissociation of the conjugate acid of TBD and 4-methylbenzyl alcohol, as well as the deprotonation of the alcohol by TBD, are shown below:

$$TBDH^+ \rightleftharpoons TBD + H^+ \qquad \text{Equation (S11)}$$

$$R\text{-}OH \rightleftharpoons R\text{-}O^- + H^+ \qquad \text{Equation (S12)}$$

$$R\text{-}OH + TBD \rightleftharpoons R\text{-}O^- + TBDH^+ \qquad \text{Equation (S13)}$$

Assuming the rate constants for Equations (S11) and (S12) are denoted as K_{a1} and K_{a2}, respectively, the equilibrium constant (K) for the deprotonation of 4-methylbenzyl alcohol by TBD (Equation (S13)) can be expressed as $K = K_{a2} / K_{a1}$.

As the pK_a values of 4-methylbenzyl alcohol and TBD in dichloromethane (DCM) are not available in the literature, acetonitrile was selected as a surrogate solvent due to its similar physicochemical characteristics. Both acetonitrile and DCM are polar aprotic solvents with limited hydrogen-bonding ability and comparable solvation behaviour. Accordingly, the pK_a values of $TBDH^+$ and 4-methylbenzyl alcohol in acetonitrile were used to estimate the equilibrium constant (K) for the deprotonation process.

$$K = \frac{K_{a2}}{K_{a1}} = \frac{10^{-pK_{a2}}}{10^{-pK_{a1}}} = 10^{pK_{a1} - pK_{a2}} = 10^{26.03 - 27.7} = 0.0214$$

With the rate constant for the deprotonation of 4-methylbenzyl alcohol by TBD being determined, the extent of alcohol deprotonated at different initial monomer concentrations and DPs was calculated and summarized in Table S24.

Table S24. Summary of the extent of 4-methylbenzyl alcohol being deprotonated by TBD at different initial monomer concentrations and DPs, monomer to catalyst ratio is 200.

DP	*Concentration of the initial initiator and deprotonated alcohol* / ($mol \cdot L^{-1}$)							
	L-lactide: 0.2 $mol \cdot L^{-1}$		L-lactide: 0.35 $mol \cdot L^{-1}$		L-lactide: 0.5 $mol \cdot L^{-1}$		L-lactide: 0.7 $mol \cdot L^{-1}$	
	Initial initiator	Deprotonated initiator	Initial initiator	Deprotonated initiator	Initial initiator	Deprotonated initiator	Initial initiator	Deprotonated initiator
50	0.00400	0.0002461	0.00700	0.00043067	0.01000	0.00061524	0.01400	0.00086134
70	0.00286	0.00021142	0.00500	0.00036981	0.00714	0.0005282	0.01000	0.00073962
90	0.00222	0.00018792	0.00389	0.00032906	0.00556	0.0004702	0.00778	0.00065812
120	0.00167	0.00016412	0.00292	0.0002871	0.00417	0.00041007	0.00583	0.00057372
150	0.00133	0.00014696	0.00233	0.00025731	0.00333	0.00036766	0.00467	0.000515

3-dimensional first-order linear plots

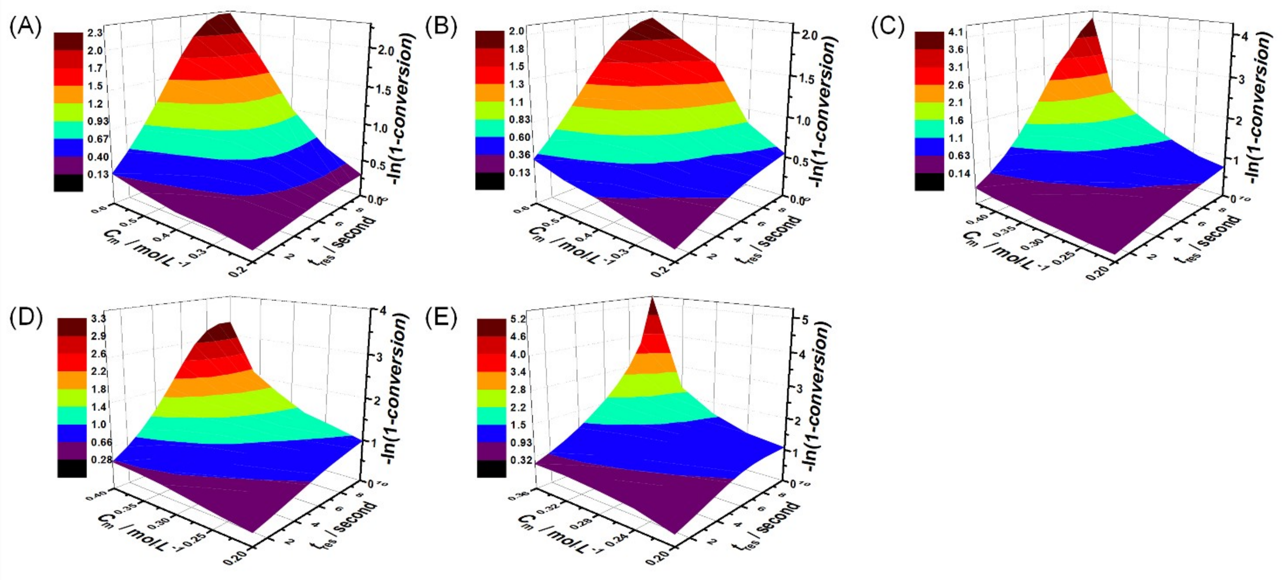

Figure S22. Plots of $\ln(M_0/M_t)$ against residence time and monomer concentration for concentration-sweep experiments conducted at (A) 35 ˚C, (B) 30 ˚C, (C) 20 ˚C, (D) 10 ˚C and (E) 0 ˚C.

Plots of the actual rate constant from different experiments

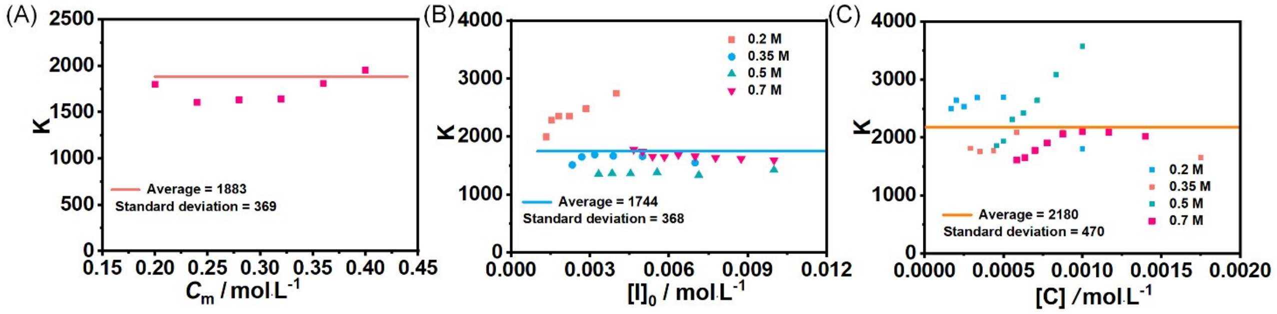

Figure S23. Plots of the corrected rate constants for the TBD-catalyzed ring-opening polymerization (ROP) of L-lactide initiated by 4-methylbenzyl alcohol at 20 °C: (A) from monomer concentration sweeping experiment, (B) from degree of polymerization sweeping experiment carried out at different initial monomer concentrations, and (C) from monomer-to-catalyst ratio sweeping experiment at different initial monomer concentrations.

4. Raw data access

All the raw data from the experiment is uploaded online. The raw data is available in a Monash research data repository at https://doi.org/10.26180/27824139.v2. All the Python scripts used are available in a GitHub repository at https://github.com/PRDMonash/FT-IR_screening_of_ROP_of_L-lactide.

Reference

1. B. Braun, J. R. Dorgan and S. F. Dec, *Macromolecules*, 2006, **39**, 9302-9310.
2. S. A. van den Berg, H. Zuilhof and T. Wennekes, *Macromolecules*, 2016, **49**, 2054-2062.
3. B. G. G. Lohmeijer, R. C. Pratt, F. Leibfarth, J. W. Logan, D. A. Long, A. P. Dove, F. Nederberg, J. Choi, C. Wade, R. M. Waymouth and J. L. Hedrick, *Macromolecules*, 2006, **39**, 8574-8583.
4. I. Kaljurand, A. Kütt, L. Sooväli, T. Rodima, V. Mäemets, I. Leito and I. A. Koppel, *The Journal of Organic Chemistry*, 2005, **70**, 1019-1028.
5. E. Rossini, A. D. Bochevarov and E. W. Knapp, *ACS Omega*, 2018, **3**, 1653-1662.
